# IL33-mediated ILC2 activation and neutrophil IL5 production in the lung response after severe trauma: A reverse translation study from a human cohort to a mouse trauma model

**DOI:** 10.1371/journal.pmed.1002365

**Published:** 2017-07-25

**Authors:** Jing Xu, Jesse Guardado, Rosemary Hoffman, Hui Xu, Rami Namas, Yoram Vodovotz, Li Xu, Mostafa Ramadan, Joshua Brown, Heth R. Turnquist, Timothy R. Billiar

**Affiliations:** 1 Department of Surgery, University of Pittsburgh, Pittsburgh, Pennsylvania, United States of America; 2 State Key Laboratory of Trauma, Burns and Combined Injury, Second Department of Research Institute of Surgery, Daping Hospital, Third Military Medical University, Chongqing, P. R. China; 3 State Key Laboratory of Oral Diseases, Department of Orthodontics, West China Hospital of Stomatology, Sichuan University, Chengdu, Sichuan, P. R. China; 4 Department of Emergency Medicine, Union Hospital, Tongji Medical College, Huazhong University of Science and Technology, Wuhan, Hubei, P. R. China; Barts and the London School of Medicine & Dentistry, Queen Mary University of London, UNITED KINGDOM

## Abstract

**Background:**

The immunosuppression and immune dysregulation that follows severe injury includes type 2 immune responses manifested by elevations in interleukin (IL) 4, IL5, and IL13 early after injury. We hypothesized that IL33, an alarmin released early after tissue injury and a known regulator of type 2 immunity, contributes to the early type 2 immune responses after systemic injury.

**Methods and findings:**

Blunt trauma patients admitted to the trauma intensive care unit of a level I trauma center were enrolled in an observational study that included frequent blood sampling. Dynamic changes in IL33 and soluble suppression of tumorigenicity 2 (sST2) levels were measured in the plasma and correlated with levels of the type 2 cytokines and nosocomial infection. Based on the observations in humans, mechanistic experiments were designed in a mouse model of resuscitated hemorrhagic shock and tissue trauma (HS/T). These experiments utilized wild-type C57BL/6 mice, IL33^-/-^ mice, B6.C3(Cg)-Rora^sg/sg^ mice deficient in group 2 innate lymphoid cells (ILC2), and C57BL/6 wild-type mice treated with anti-IL5 antibody.

Severely injured human blunt trauma patients (*n* = 472, average injury severity score [ISS] = 20.2) exhibited elevations in plasma IL33 levels upon admission and over time that correlated positively with increases in IL4, IL5, and IL13 (*P* < 0.0001). sST2 levels also increased after injury but in a delayed manner compared with IL33. The increases in IL33 and sST2 were significantly greater in patients that developed nosocomial infection and organ dysfunction than similarly injured patients that did not (*P* < 0.05). Mechanistic studies were carried out in a mouse model of HS/T that recapitulated the early increase in IL33 and delayed increase in sST2 in the plasma (*P* < 0.005). These studies identified a pathway where IL33 induces ILC2 activation in the lung within hours of HS/T. ILC2 IL5 up-regulation induces further IL5 expression by CXCR2^+^ lung neutrophils, culminating in early lung injury. The major limitations of this study are the descriptive nature of the human study component and the impact of the potential differences between human and mouse immune responses to polytrauma. Also, the studies performed did not permit us to make conclusions about the impact of IL33 on pulmonary function.

**Conclusions:**

These results suggest that IL33 may initiate early detrimental type 2 immune responses after trauma through ILC2 regulation of neutrophil IL5 production. This IL33–ILC2–IL5–neutrophil axis defines a novel regulatory role for ILC2 in acute lung injury that could be targeted in trauma patients prone to early lung dysfunction.

## Abstract 2

In a reverse translation study from a human cohort to a mouse trauma model, Timothy Billiar and colleagues investigate the factors mediating immune dysregulation after severe injury.

## Author summary

**Why was this study done?:**

- The factors that initiate immune dysregulation after severe injury, including the skewing of the immune system towards type 2 responses, are unknown.
- Our aim was to determine if interleukin (IL) 33, an alarmin released after tissue injury and a regulator of type 2 immunity, contributes to the early type 2 immune responses after systemic injury.

**What did the researchers do and find?:**

- We measured IL33 and its soluble receptor, soluble suppression of tumorigenicity 2 (sST2), an endogenous antagonist of IL33, in the blood of 472 multiply injured trauma patients.
- IL33 levels were already markedly elevated on the first blood draw after injury and correlated with increases in IL4, IL5, and IL13 as well as nosocomial infections.
- Reverse translation experiments using a mouse model of hemorrhagic shock and tissue trauma were carried out to understand the functional link between IL33 and the type 2 cytokine, IL5.
- IL33 was shown to activate a resident set of innate lymphocytes (group 2 innate lymphoid cells [ILC2]) in the lungs to produce IL5, further leading to neutrophil IL5 production.

**What do these findings mean?:**

- These findings demonstrate the utility of using reverse translation of observations made in humans into mechanistic experimental models that recapitulate key components of the clinical response.
- IL33 and one of its target cells, ILC2, are identified for the first time as regulators of the early type 2 immune responses after severe injury.
- The findings include a previously unsuspected role for ILC2 in the regulation of neutrophils and acute lung injury.
- The findings identify IL33, IL5, or their receptors as potential targets to block early lung injury after polytrauma.

## Introduction

Severe traumatic injury is a leading cause of death and morbidity in humans between the ages of 1 and 44 years [1,2]. Recent advances in treatments targeting blood loss and coagulopathy have markedly reduced early deaths. However, secondary complications, including acute respiratory distress syndrome (ARDS), multiple organ dysfunction syndrome (MODS), and nosocomial infections (NIs) remain significant causes of morbidity in hospitalized trauma patients [3,4]. In patients that progress to organ dysfunction, the lung is the first organ to fail [5,6] as well as being the most common site for NI [7] after severe injury. Although small-animal preclinical models do not mimic all aspects of the complex human response to severe injury [8], reverse translation of novel observations made in humans in well-characterized experimental models has been proposed as an efficient way to gain insights into the mechanisms of immune dysfunction after severe injury [9].

Organ failure following trauma has been linked to the magnitude of the early surge of cytokines, chemokines, and damage-associated molecular pattern (DAMP) molecules [10], while NI rates are associated with multiple organ failure (MOF) [11,12]. We have recently provided evidence that trauma patients that go on to develop NI also have higher levels of type 2 cytokines, including interleukin (IL) 4, IL5, and IL13 within 24 hours after injury [7]. The conversion towards type 2 responses after trauma has been most frequently attributed to a shift in the balance between Th1 and Th2 cells [13–17]. However, polarization of naïve T cells can take several days [15,17], so the early appearance of type 2 cytokines in the circulation after injury suggests that other, more rapid response pathways account for the dynamics of the injury-induced type 2 response.

Tissue damage can lead to the release of self-derived “danger” signals, or alarmins, that alert the immune system to potential breeches in barrier integrity [18–20]. IL33, a member of the IL1 cytokine superfamily, is constitutively expressed in the epithelium of both mice and humans and acts as an alarmin after tissue injury by activating immune cells through its cognate receptor, suppression of tumorigenicity (ST2) [21–23]. Group 2 innate lymphoid cells (ILC2) are a recently described subset of linage-negative ST2^+^, GATA3-dependent lymphocytes found in the subepithelial space in the lungs and other barrier organs as well as adipose tissue [24]. ILC2 are noncytolytic but can be activated to produce the type 2 cytokines IL5 and IL13 in response to IL33 and other signals from the adjacent epithelium [24,25]. ILC2 have been shown to be important in the clearance of helminth infections, in the promotion of allergic airway inflammation, and in epithelial repair [26–28]. An important, unanswered question remains: are ILC2 stimulated by IL33 released during traumatic injury to support potentially detrimental type 2 responses?

In the present study, we made observations in a cohort of severely injured blunt trauma patients and reverse translated these observations in a well-described mouse polytrauma model using C57BL/6 mice, IL33^-/-^ mice, and ILC2-deficient B6.C3(Cg)-Rora^sg/sg^ mice. We tested the hypothesis that IL33 drives an early type 2 immune response in the lung through the activation of resident ILC2.

## Methods

### Ethical approval

All human sampling was carried out after approval by the University of Pittsburgh institutional review board and in compliance with the STROBE guidelines at http://www.equator-network.org/reporting-guidelines/strobe/ (S1 Text). Written informed consent within 48 hours of presentation was obtained from all participants. A prospective analysis plan was submitted as part of our IRB application in November 2015 (S2 Text). All the animal experimental protocols were approved by the Institutional Animal Care and Use Committee of the University of Pittsburgh and complied with the *Guide for the Care and Use of Laboratory Animals* (published by the United States National Institutes of Health) and the ARRIVE guidelines at http://www.nc3rs.org.uk/arrive-guidelines (S3 Text).

### Reagents

Anti-mouse CD45 AF700 (30-F11), fixable viability dye eFluor 506, anti-mouse CD45R FITC (RA3-6B2), anti-mouse CD11b FITC (M1/70), anti-mouse CD25 APC (PC61.5), anti-mouse Ly-6A/E (Sca-1) PE (D7), anti-mouse CD3e FITC (145-2C11), anti-mouse CD25 PE (PC61.5), anti-mouse CD117 PE Cy7 (ACK2), anti-mouse CD90.2 APC eFluor 780 (53–2.1), anti-mouse CD127 eFluor 450 (A7R34), anti-mouse ST2 PercP eFluor 710 (RMST2-2), anti-mouse F4/80 PE (BM8), anti-mouse CD11C PE-Cy7 (N418), anti-mouse Ly6 C Percp cy5.5 (HK1.4), anti-mouse CD11b PE-eFluor 610 (M1/70), anti-mouse CD45R PE eF610 (RA3-6B2), anti-mouse CD11b eF450 (M1/70), anti-mouse CD45.2 AF700 (104), anti-mouse CD45.1 PE-eFluor 610 (A20), anti-mouse ST2 (IL33R) APC (RMST2-2), anti-mouse CD125 BV421 (T21), OneComp eBead, and Foxp3/transcription factor staining buffer were from eBioscience. Ionomycin, PMA, nuclear staining Bisbenzimide H 33258, Histopaque 1077, and Histopaque 1191 were from Sigma. Anti-mouse CXCR2 PE (242216); immunoassay (ELISA) kit for mouse IL33, IL5, and ST2; anti-IL33 goat IgG; and recombinant mouse IL5 were from R&D Systems (P04401). Anti-mouse NK1.1 FITC (PK136); anti-mouse GATA3 PE-CF594 (L50-823) and PE-CF594 mouse IgG1, κ isotype control (X40); anti-mouse IL5 APC (TRFK5) and APC Rat IgG1, κ isotype control (R3-34); anti-mouse Ly6G APC Cy7 (1A8); anti-mouse NK1.1 APC (PK136); anti-mouse CD11b PE (M1/70); protein transport inhibitor; purified anti-mouse CD16/CD32 (mouse BD Fc block, 2.4G2); purified NA/LE anti-mouse IL5 (TRFK5); and purified NA/LE rat IgG1 κ isotype control (R3-34) were from BD Biosciences. Anti–collagen I rabbit IgG, and anti-CD45 rat IgG were from Abcam. Alexa Cy5–conjugated donkey anti-goat IgG, Alexa Cy3–conjugated donkey anti-rat IgG, and Alexa 488–conjugated donkey anti-rabbit IgG were from Jackson ImmunoResearch. Lung dissociation kit (mouse), anti-Ly6G microbead kit, and MACS LS columns were from Miltenyi Biotec. RT^2^ qPCR primer assay for mouse IL5 (NM_010558) and beta actin (NM_007393), Qia shredder, and RNeasy mini kit were from Qiagen. 5× iScript RT supermix and iTaq universal SYBR Green supermix were from Bio-rad. RPMI 1640 (PBS) was from Lonza Bio Whittaker. 10× cell lysis buffer was from Cell Signaling Technology. Immunoassays, MILLIPLEX map kit for human IL33, IL4, IL5, and IL13, and Luminex multiple assay were from EMD Millipore.

### Trauma patient enrollment and data collection

Blunt trauma patients presenting between the ages of 18 and 90 years old and admitted to the trauma intensive care unit were eligible for enrollment in this observational study. Written informed consent within 48 hours of presentation was obtained from all participants. Exclusion criteria included isolated head or brain injury, being unlikely to survive beyond 24 hours, and pregnancy. These criteria were chosen based on the goals of correlating early biomarker patterns with clinical outcomes over several days after systemic injury because of blunt trauma. All eligible patients were enrolled with the exception of patients presenting during time periods that research staff were not available to screen patients and obtain plasma samples. For this analysis, we also excluded 21 (out of 493) patients that died during the initial hospital stay based on the goal of measuring plasma biomarkers over a minimum of the first 7 days. The 472 blunt trauma survivors that had an average ISS of 20.2 were studied in a retrospective analysis. Plasma samples were drawn upon admission and then at 2 additional time points in the first 24 hours as well as daily for the first week. IL33 and type 2 cytokines (IL4, IL5, and IL13) were measure by Luminex multiplex assay and the soluble form of ST2, soluble ST2 (sST2) by ELISA.

### Animals

C57BL/6 male mice, B6.C3 (Cg)-Rora^sg/^J mice, and the B6.SJL-Ptprc Pepc male mice were purchased from Jackson Laboratories. B6 IL33^-/-^ mice were originally provided by Dr. S. Nakae [29] and maintained in our animal facility. All mice were housed in the animal research center according to National Institutes of Health (NIH) animal care guidelines, with the 12:12 hour light–dark cycle and free access to standard laboratory food and water.

### Mouse model of HS/T

Mice, age 8–12 weeks and weighing 20–30 g, were randomized into different experimental groups and anesthetized with pentobarbital sodium (80 mg/kg, i.p.), with repeated doses (10 mg/kg, i.p.) given when necessary during the experiment. The HS/T model was performed as previously described by our group [30,31]. First, crushed bone fragments were prepared from humanely killed donor mice using the femurs and tibias of both hind limbs. The fragments were crushed into bone a mixture in a 2 ml PBS using a sterile mortar and pestle. Additional mice were anesthetized and their limbs fixed against a warm operation plate to avoid low body temperature and groin incisions were performed. The femoral arteries of both hind limbs were cannulated with a sterile PE-10 catheter, 1 for bleeding, and the other was connected with a blood pressure monitor to record mean arterial pressure (MAP). Then both thighs were crushed for 30 seconds with a hemostat to induce soft tissue lesion, and the bone mixture from the donor mice was injected into the soft tissue injury area (0.15 ml/each thigh) to induce a pseudofracture (PF) injury. Subsequently, blood was withdrawn through femoral artery cannula to induce hemorrhagic shock (HS); the MAP was maintained at 25 mmHg. After 2 hours of HS/T, animals were resuscitated with Ringer’s lactate solution at 3 times the volume of shed blood. Mice were alive, kept warm with access to food and water after the HS/T model, and then humanely killed at 6 hours, 24 hours, 48 hours, or 72 hours following the initiation of resuscitation. The control group for the polytrauma mice was uninjured mice. This permitted us to compare baseline parameters (i.e., the preinjury state) to the accumulated impact of all of the acute insults commonly experienced by polytrauma patients, which include (1) vessel cannulation, (2) hemorrhagic shock, (3) severe tissue trauma, and (4) anesthesia plus pain control. We have previously shown that the impact of these insults is indeed cumulative at the level of mRNA expression and the systemic inflammatory response [32,33].

For IL5 neutralization, mice received 2 purified NA/LE anti-mouse IL5 tail vein injections (50 μg/mouse), including 30 minutes before completing the HS/T model and then immediately before the resuscitation. In the isotype control group, a purified NA/LE rat IgG1 κ isotype was injected instead.

### Lung tissue dissociation, cell isolation, and flow cytometric analysis

Mice were euthanized at the indicated time points after HS/T and their lungs were removed and dissociated into a single-cell suspension with a lung dissociation kit. Single cells were incubated with fixable viability dye eFluor 506 for dead cells exclusion, incubated with purified anti-mouse CD16/CD32 to block Fc receptors, and then stained with different fluorescently labeled monoclonal antibodies (mAb). For ILC2 detection (surface staining), FITC-conjugated lineage marker mAbs (CD3e, CD11b, CD45R, and NK1.1), anti-mouse CD45 AF700, anti-mouse CD25 APC, anti-mouse Ly-6A/E (Sca-1) PE, anti-mouse CD117 PE Cy7, anti-mouse CD90.2 APC eFluor 780, anti-mouse CD127 eFluor 450, and anti-mouse ST2 PercP eFluor 710 were used [34]. For ILC2 IL5 intercellular staining, FITC-conjugated lineage marker mAbs (CD3e, CD11b, CD45R, and NK1.1); anti-mouse CD45 AF700; anti-mouse CD25 PE; anti-mouse CD127 eFluor 450; anti-mouse GATA3 PE-CF594; PE-CF594 mouse IgG1, κ isotype control; anti-mouse IL5 APC; and APC Rat IgG1, κ isotype control were used. For neutrophil IL5 detection, anti-mouse CD45 AF700, anti-mouse CD11b PE-eFluor 610, anti-mouse Ly6G APC Cy7, anti-mouse CXCR2 PE, anti-mouse IL5 APC, and APC Rat IgG1, κ isotype control were used. For the lung leukocyte phenotype detection, anti-mouse CD45 AF700, anti-mouse CD11b eF450, anti-mouse F4/80 PE, anti-mouse CD11C PE-Cy7, anti-mouse Ly6 C Percp cy5.5, anti-mouse Ly6G APC Cy7, anti-mouse CD45R PE eF610, anti-mouse CD3e FITC, and anti-mouse NK1.1 APC were used. For neutrophil receptors detection, anti-mouse ST2 (IL33R) APC and anti-mouse CD125 BV421 were used. To detect intracellular IL5, PMA (10 ng/ml), ionomycin (500 ng/ml), and protein transport inhibitor (1 μl/ml) were used to stimulate cells for 3 hours before staining, and Foxp3/transcription factor staining buffer was used for permeabilization and fix. OneComp eBeads were used for the subclass control. A BD STI LSR II flow cytometer was used for signal detection and collection; Flowjo V10.1 was used for data analysis.

### Bone marrow transplantation (BMT) and ILC2 deletion in mice

Based on previous research [35], B6.C3(Cg)-Rora^sg/^J mice were bred to use the wild-type mice (WT) (Rora^+/+^) and ILC2-deficienct mice (Rora^sg/sg^) as the BMT donors. B6.SJL-Ptprc Pepc mice were lethally irradiated at 1,000 Rads and subsequently received intravenous transplantation of 10^7^ whole bone marrow cells from WT (Rora^+/+^) or Rora^sg/sg^ mice 2–3 weeks old, were given an antibiotic diet (sulfamethoxazole and trimethoprim) for 2 weeks, and were used for the HS/T model 12 weeks after transplantation. Before making the HS/T model, about 100 μl of blood was collected from orbital sinus to identify the efficiency of BMT using the anti-mouse CD45.2 AF700 and anti-mouse CD45.1 PE-eFluor 610.

### Neutrophil isolation, culture, and IL5 detection by PCR and flow cytometry

To assess lung neutrophil IL5 mRNA levels in response to exogenous IL5, neutrophils were isolated from lung single-cell suspensions with the anti-Ly6G microbead kit and MACS manual separator (Miltenyi Biotec). The neutrophil mRNA was extracted using RNeasy Mini Kit and RNA reverse transcribed with iScript RT supermix. PCR amplification to detect IL5 mRNA expression was performed in an iTaq Universal SYBR Green Supermix using a Bio-Rad real-time system.

To assess with flow, neutrophils were isolated from mouse bone marrow as described [36]. Briefly, bone marrow cells were harvested from mouse femurs and tibias; 3 ml Histopaque 1119 was added in a 15 ml conical centrifuge tube and overlaid with 3 ml of Histopaque 1077, and this overlaid the bone marrow cell suspension. This was centrifuged at 872 × g at room temperature for 30 minutes. Neutrophils were collected from the interface of the Histopaque 1077 and Histopaque 1199 layers, then washed with RPMI 1640 (supplemented with 10% FBS) and centrifuged at 427 × g for 5 minutes at 4°C. 2 ng/ml or 10 ng/ml recombinant mouse IL5 was added into the separated neutrophils for 15 minutes, then PMA (10 ng/ml), ionomycin (500 ng/ml), and protein transport inhibitor (1 μl/ml) were added at 37°C for 3 hours, and neutrophil IL5 was detected by intracellular staining and flow cytometry.

### Rodent IL33, IL5, and sST2 measurements

For lung IL33- and IL5-level detection, lung tissue was removed from euthanized mice at the indicated time points after HS/T. The lung protein lysis supernatant was harvested using cell lysis buffer. After the protein concentration measurement and adjustment, IL33 levels were measured by ELISA according to manufacturer’s instructions, with the protein concentration adjusted to 50 μg/sample and the protein concentration for IL5 detection at 500 μg/sample. For plasma IL33, IL5, and sST2 level detection, blood was obtained from euthanized mice by cardiac puncture and anticoagulated with heparin sodium. Plasma was collected by centrifuging the anticoagulated whole blood at 300 × g for 10 minutes at 4°C, then IL33, IL5, and soluble ST2 levels in plasma were detected by ELISA according to manufacturer’s instructions.

### Lung histologic studies

For immunofluorescence staining, the right upper lobes of the lung were inflated, followed by perfusion fixation with PBS and then 2% paraformaldehyde. Tissue was then placed in 2% paraformaldehyde for an additional 2 hours and then switched to 30% sucrose in a distilled water solution for 12 hours. The tissue was then slowly frozen in 2-methylbutane according to a standardized protocol for cryopreservation [37]. Tissue sections (5 μm) were incubated with 2% bovine serum albumin (BSA) in PBS for 1 hour, followed by 5 washes with PBS containing 0.5% BSA (PBB). The samples were then incubated overnight with primary antibodies. The following primary antibodies were used for staining: anti-IL33 (5 μg/ml + 0.01% Triton X-100 in PBB), anti–collagen I (2 μg/ml in PBB), and anti-CD45 (2 μg/ml in PBB). Alexa Cy5–conjugated donkey anti-goat IgG (1:1,000 for anti-IL33 antibody, shown in white), Alexa Cy3–conjugated donkey anti-rat IgG (1:500 for anti-CD45 antibody, shown in green), and Alexa 488–conjugated donkey anti-rabbit IgG (1:500 for anti–Collagen I antibody, shown in red) were used as secondary antibodies. Nuclear staining was carried out with Bisbenzimide H 33258 (20 μmol/L, shown in blue). Imaging conditions were maintained at identical settings within each antibody-labeling experiment, with original gating performed using the negative control. Imaging was performed with 400× magnification using a Nikon A1 confocal microscope (Nikon, Melville, NY). Quantification was performed using NIS Elements (Nikon, Melville, NY).

Right upper lung lobes processed as described above were stained for hematoxylin and eosin (H&E) to evaluate histopathologic cumulative changes among treatment groups [30]. Images of 5 randomly selected fields were acquired using an Olympus Provis light microscope (Malvern, NY) with 200× magnification. Histopathologic changes were assessed by an observer blinded to experimental treatment using the following features: congestion, hemorrhage, edema, and cellular infiltration. The lung injury degree was quantitatively graded on a scale from 0 to 4 for each feature (0, normal; 1, light; 2, moderate; 3, severe; and 4, very severe), and the maximum possible score was 16.

### Statistics

The results presented in the study are expressed as the mean ± SEM. Statistical differences between the 2 groups were established by unpaired 2-tailed Student *t* test. Group–time interactions of plasma inflammatory mediator levels in patient groups were compared by 2-way ANOVA. Correlations were measured with the pairwise Pearson correlations test using GraphPad Prism 6 software (GraphPad Software, San Diego, CA). In all studies, differences were considered significant when *P* < 0.05.

Data were deposited in the Dryad repository: http://dx.doi.org/10.5061/dryad.kt8m4 [38].

## Results

### Elevated plasma IL33 early after trauma correlates with clinical complications

**Fig 1:**
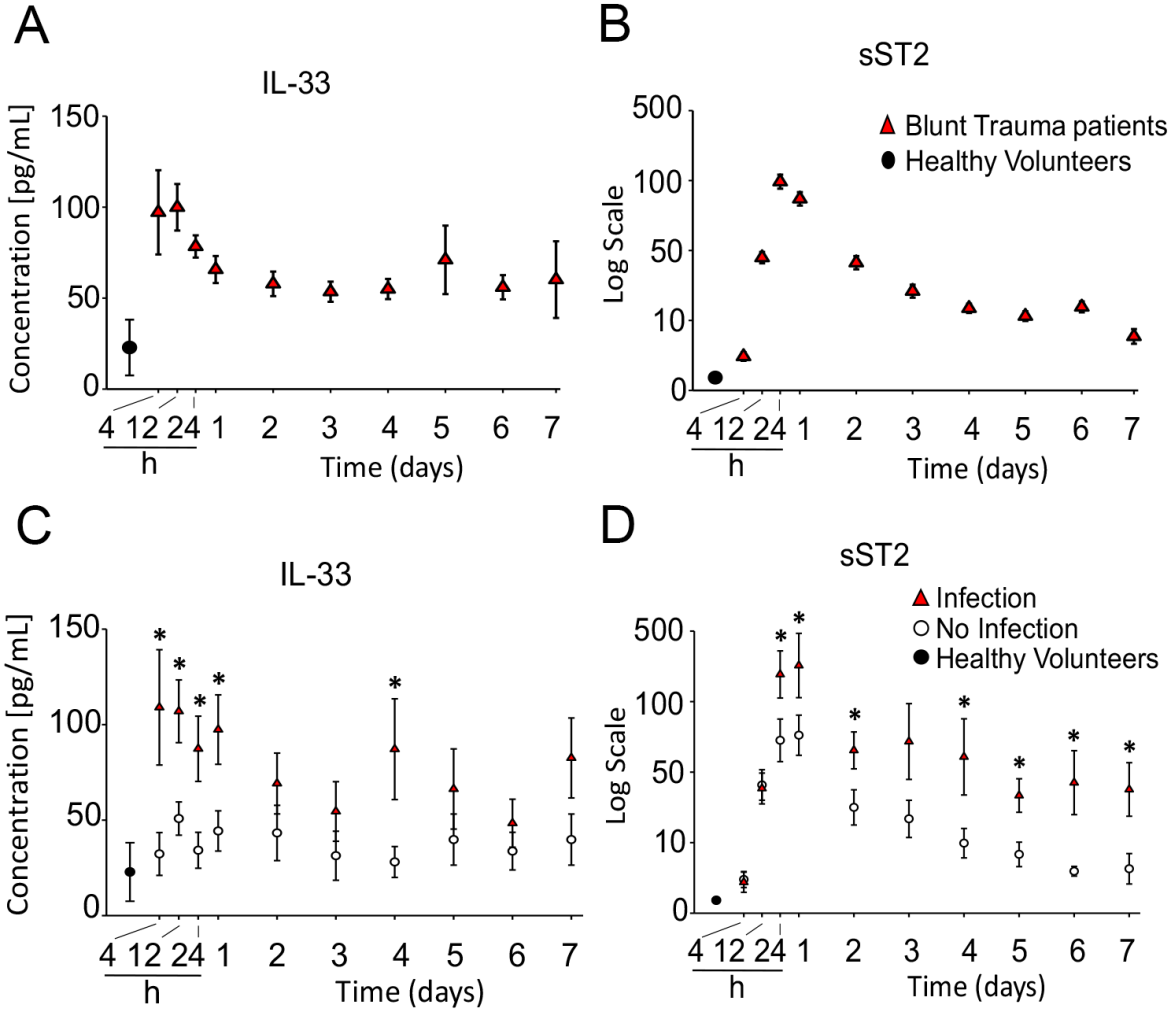
Time course analysis of mean circulating levels of interleukin (IL) 33 and soluble suppression of tumorigenicity (sST2) in blunt trauma patients. The inflammatory mediators were assessed in serial plasma samples obtained at the indicated time points, starting with the first blood draw upon hospital admission and followed by 2 blood draws within the first 24 hours after injury and then daily thereafter up to day 7. A: Time course of IL33 in trauma patients (*n* = 472 survivors) versus healthy volunteers (HVs) (*n* = 12). B: Time course of sST2 in trauma patients (*n* = 472) versus HVs (*n* = 12). C: Time course analysis of mean circulating levels of IL33 in trauma patients diagnosed with nosocomial infection (NI) (*n* = 44) versus patients without NI (*n* = 44) matched for age, sex, and injury severity. D: Time course analysis of mean circulating levels of sST2 in trauma patients with NI (*n* = 44) versus patients without NI (*n* = 44). * *P* < 0.05.

IL33 is described as an alarmin that is released after tissue injury; however, the patterns of IL33 release in humans experiencing multiple trauma have not been determined. We measured circulating IL33 levels over time in 472 blunt trauma patients admitted to our trauma intensive care unit (ICU). The demographic and clinical characteristics of these patients have been described previously in detail (7). Briefly, the subjects had an average age of 42 (range 18 to 90), were ~70% male, and had an average ISS of 20.2 (range 1 to 50). Blood samples were obtained upon admission, at 2 additional time points in the first 24 hours, and then daily until day 7 or discharge from the ICU. Compared to IL33 levels obtained in healthy volunteers (*n* = 12), trauma patients exhibited significant elevations in IL33 levels at admission (Fig 1A). In our study, the healthy volunteers (*n* = 12) had an average baseline IL33 level of 22.8 pg/mL. This is midrange (5–53 pg/mL) for what has been reported in healthy adult humans [39–43]. In injured patients, IL33 levels were the highest in the first 4 hours after presentation, and this was followed by persistent elevations through 7 days in patients that remained in the ICU. IL33 levels did not correlate with ISS and there were no differences in the average levels between female and male subjects (S1 and S2 Figs).

The soluble form of the IL33 cell surface receptor, sST2, acts as endogenous inhibitor of the extracellular functions of IL33 [44] and is known to be elevated during pathological inflammatory states [18,45,46]. Plasma sST2 displayed significant elevatation early after polytrauma and exhibited sustained increases over the next 7 days (Fig 1B). Relative to the increases in IL33, however, peak elevations in sST2 levels were delayed by 12–24 hours (Fig 1A versus 1B).

Negative outcomes associated with immune dysfunction after injury include MODS, ARDS, and increased susceptibility to NI [47]. Accordingly, we next determined if IL33 levels were different in patients that developed NI compared to patients that did not. This was done by comparing cohorts with or without NI (*n* = 44 each) matched for injury severity (based on ISS), age, and sex to exclude potential confounding based on differences in these variables (S1 Table). NI was diagnosed on day 6 on average, and we have previously reported that patients in the NI cohort also experience significantly higher incidences of other adverse in-hospital outcomes, including prolonged respiratory failure, higher MODS scores, and longer intensive care unit length of stay [7]. The NI group had significantly higher circulating IL33 levels on admission and over time (Fig 1C). While this group also had higher circulating sST2 levels, unlike IL33, which was highest at admission, sST2 levels did not peak until 12–24 hours posttrauma (Fig 1D). These data suggest that high levels of IL33 released early posttrauma may contribute to negative outcomes after trauma.

### IL33 levels correlate with type 2 cytokines in humans following trauma

A skewing towards a type 2 immune response is thought to contribute to impaired immune defenses in severely injured humans [48]. We have previously shown that trauma patients that develop NI have higher IL4, IL5, and IL13 levels within 24 hours of injury [7]. We sought to determine if early elevations in IL33 levels correlated with increases in blood levels of type 2 cytokines. As shown in Table 1, IL33 levels upon admission, at 24 hours, and at days 2–3 correlated significantly with increases in IL4, IL5, and IL13 at the same time points. However, IL33 did not show a positive correlation with circulating levels of IL6, an inflammatory mediator well known to be increased in injured humans [7,49,50] but not associated with type 2 immune responses. Interestingly, increases in sST2 levels did not correlate with the type 2 cytokines but did correlate with IL6 levels at all time points over the first 3 days.

**Table 1:** Correlations of circulating interleukin (IL) 33 and soluble suppression of tumorigenicity (sST2) with type 2 cytokines (IL4, IL5, and IL13) and IL6 in blunt trauma patients (*n* = 472, all survivors) upon admission, at 24 hours, and days 2 to 3 postinjury.

	IL4	IL5	IL13	IL6
***Admission***				
**IL33**	CC = 0.9	CC = 0.8	CC = 0.9	CC = 0.07
***P* value**	<0.0001	<0.0001	<0.0001	0.321
**sST2**	CC = –0.03	CC = –0.04	CC = –0.03	CC = 0.3
***P* value**	0.725	0.581	0.662	0.0003
***At 24 hours***				
**IL33**	CC = 0.2	CC = 0.1	CC = 0.1	CC = 0.005
***P* value**	<0.0001	<0.0001	<0.0001	0.865
**sST2**	CC = 0.04	CC = 0.04	CC = 0.02	CC = 0.3
***P* value**	0.150	0.177	0.410	<0.0001
***Days 2 to 3***				
**IL33**	CC = 0.2	CC = 0.1	CC = 0.2	CC = 0.08
***P* value**	<0.0001	0.005	<0.0001	0.083
**sST2**	CC = –0.03	CC = –0.005	CC = 0.03	CC = 0.4
***P* value**	0.546	0.903	0.437	<0.0001

CC, correlation coefficient. Significance set at *P* < 0.05.

Using multivariable regression analyses controlling for age, sex, or ISS, we found similar significant correlations between IL33 and type 2 cytokines independent of these variables (*P* < 0.05). Taken together, the human analyses show that IL33 is elevated in severely injured humans as early as the time of presentation to a trauma center and that elevations in circulating IL33 correlated with complications and circulating type 2 cytokine levels.

### IL33 and IL5 are increased early in lung and plasma of mice subjected to HS/T

**Fig 2:**
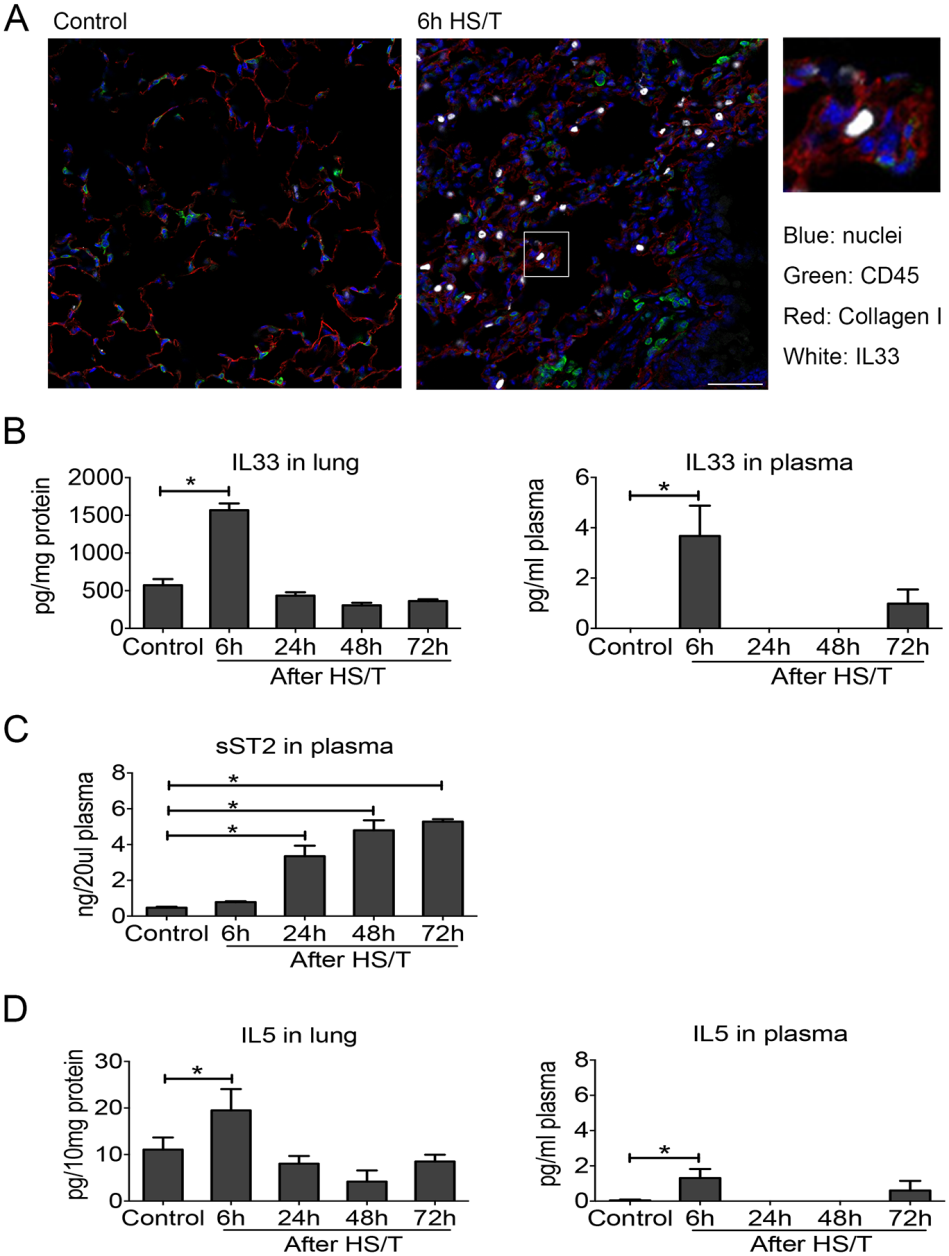
Change of interleukin (IL) 33 and type 2 cytokine IL5 in lung and plasma of mice after resuscitated hemorrhagic shock and tissue trauma (HS/T). A: Immunofluorescence of IL33 showed the increased expression of IL33 in the lungs of C57BL/6 mice after HS/T and was located in the non-CD45^+^ cells (nuclei [blue staining], CD45 [green staining], Collagen I [red staining], and IL33 [white staining]). Collagen I was used to show the lung structure. 400× magnification, scale bar 50 μm. B: IL33 was increased in the lung after HS/T, with the highest levels at 6 hours (*n* = 4–8/group), and also showed the increase at 6 hours after HS/T in plasma (*n* = 3/group, by ELISA). C: IL33 antagonist soluble suppression of tumorigenicity level in plasma was gradually increased 24 hours after HS/T (*n* = 3–4/group, by ELISA). D: IL5 was increased in the lung after HS/T, with the highest levels at 6 hours (*n* = 4–8/group), and also showed the increase at 6 hours after HS/T in plasma (*n* = 3–8/group, by ELISA). * *P* < 0.05.

To determine the mechanistic link between IL33 and type 2 cytokine production after injury, we utilized a well-characterized mouse model of HS/T [30,31]. We have shown that this model of severe injury recapitulates the early escalation of inflammation that follows systemic injury in humans [51,52] and involves an injury-induced inflammatory response that promotes organ damage and dysfunction [30,31,53]. Immunohistochemistry on samples generated using this model detected augmented cellular expression of IL33 in CD45^-^ cells in the lungs at 6 hours after HS/T (Fig 2A). There also was a profound and rapid spike (within 6 hours) of IL33 in the blood and lung after HS/T in WT C57BL/6 mice (Fig 2B). This was consistent with our clinical observations (Fig 1A). Circulating levels of sST2 after HS/T also reflected our clinical observations, as sST2 levels were not elevated in the circulation of mice at 6 hours post-HS/T but were significantly elevated at 24–72-hour time points (Fig 2C). IL5 also increased with IL33, as significant elevations in IL5 were detected in lung homogenates and in the blood at 6 hours after HS/T (Fig 2D) but then decreased to baseline by 24 hours. Thus, after severe trauma in both the mouse and human, rapid increases in IL33 are correlated with increases in the type 2 cytokine IL5.

### Neutrophils as a dominant source of early IL5 after HS/T

**Fig 3:**
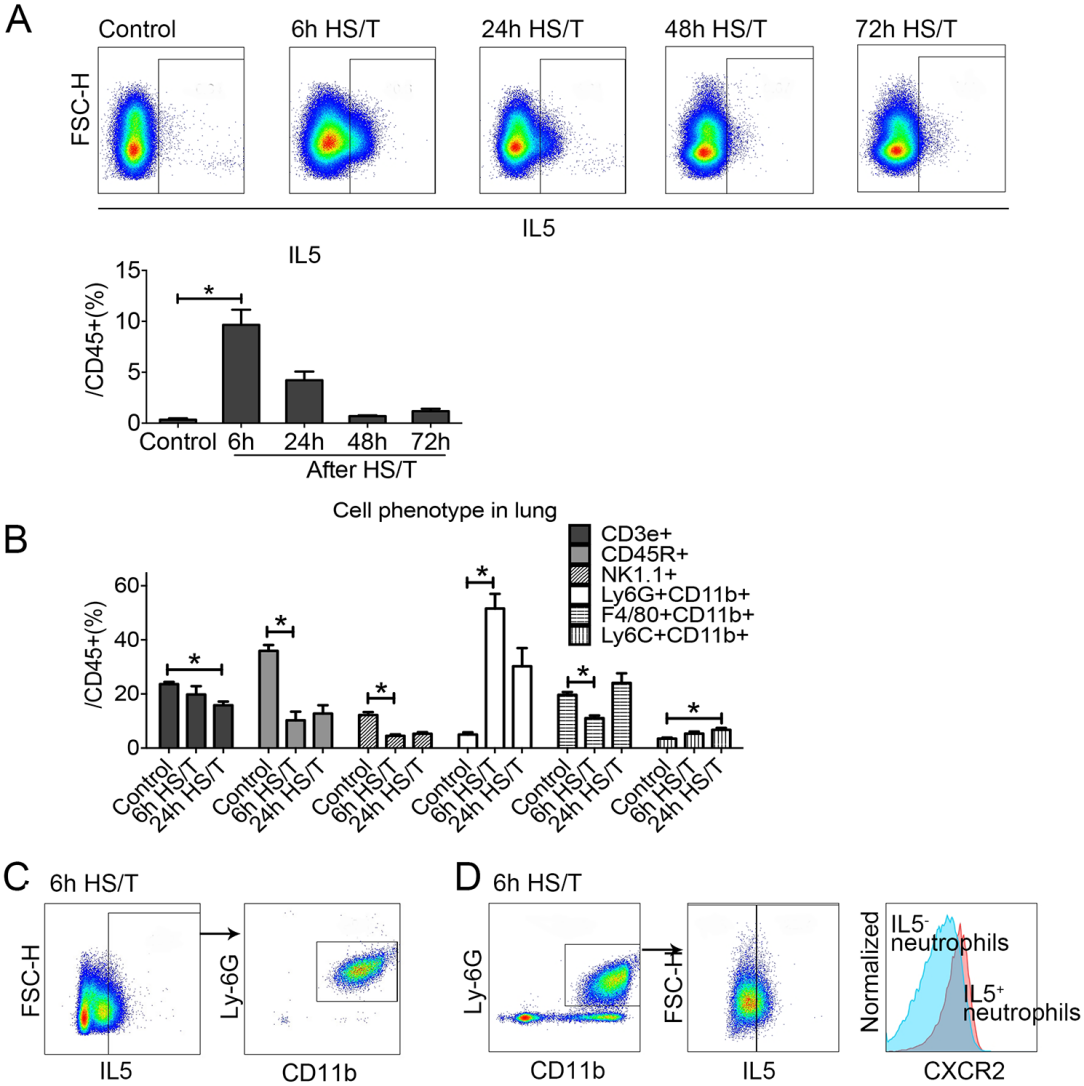
Change of cell phenotype in the lungs of mice after resuscitated hemorrhagic shock and tissue trauma (HS/T) and the cell resource of type 2 cytokine interleukin (IL) 5. A: IL5^+^ gate from lung CD45^+^ cells was increased in the lung after HS/T, peaked at 6 hours after HS/T (*n* = 5–9/group). B: The percentage of Neutrophils (Ly6G^+^CD11b^+^ gated from CD45^+^ cells) in the lungs was increased after HS/T and peaked at 6 hours, while the frequency of T cells (CD45^+^CD3e^+^), B cells (CD45^+^CD45R^+^), and natural killer (NK) cells (CD45^+^NK1.1^+^) gradually decreased in the lung after HS/T (*n* = 5–8/group). C: IL5 in the lung after HS/T was observed mostly in the neutrophils (Ly6G^+^CD11b^+^). D: IL5^+^ neutrophils in the lung had higher CXCR2^+^ expression compared with the IL5^-^ population (see histogram: the right peak shows the IL5^+^ neutrophils and the left peak shows the IL5^-^ subset). * *P* < 0.05. FSC-H, forward scatter-height.

We next determined the sources of IL5 in the lungs after HS/T. Lungs were flushed with normal saline to remove intravascular cells and leukocytes and then isolated and subjected to flow cytometry. The percentage of IL5^+^ (gated as IL5^+^CD45^+^) cells peaked at 6 hours (*P* < 0.05) and then declined gradually (Fig 3A). We then characterized the change in leukocyte populations in the lung over time after HS/T. The percentage of the CD45^+^ cells that were neutrophils (Ly6G^+^CD11b^+^ gated from CD45^+^ cells) increased after HS/T and also peaked at 6 hours (*P* < 0.05). The percentage of T cells (CD45^+^CD3e^+^), B cells (CD45^+^CD45R^+^), and natural killer (NK) cells (CD45^+^NK1.1^+^) gradually decreased in the lungs of C57BL/6 mice after HS/T (Fig 3B). Polarization of CD4 cells towards Th2 cells can be an important source of IL5; however, we found that IL5 6 hours after HS/T was predominantly from neutrophils (Fig 3C). These IL5^+^ neutrophils had higher CXCR2 expression compared with the IL5^-^ neutrophils (Fig 3D).

IL5 is typically associated with eosinophil recruitment and activation [54]; however, we found no increases in the already low numbers of cells with eosinophil markers in the lung after HS/T (S3 Fig). No roles for eosinophils have been reported in acute lung injury, and we found no change in the abundance of eosinophils in the circulation of severely injured humans based on white blood cell count differentials; the percentage of the eosinophils in the white blood cell counts remained within the normal ranges.

### ILC2 increased in the lung after HS/T and are another source of IL5

**Fig 4:**
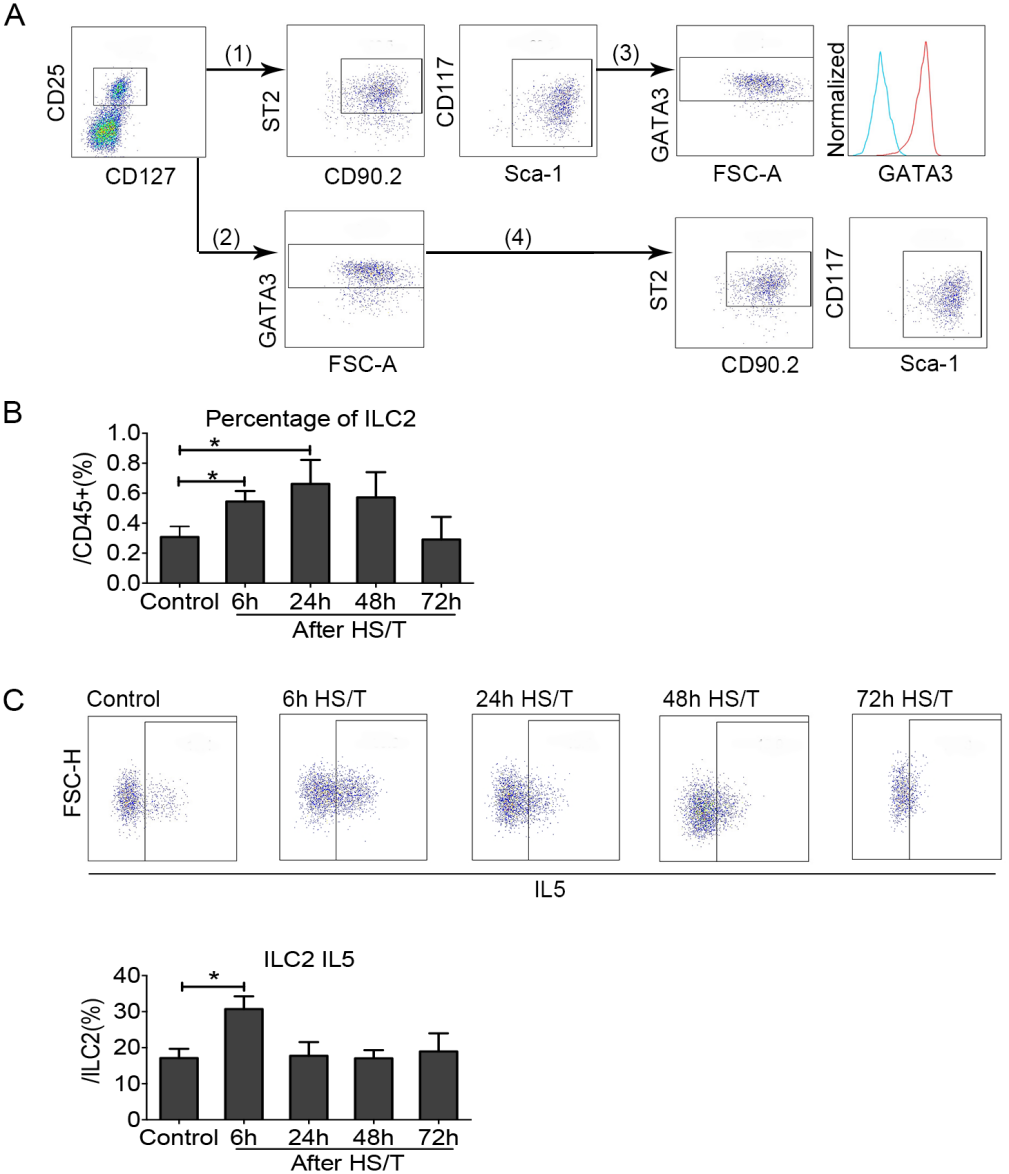
Change of group 2 innate lymphoid cells (ILC2) in the lungs of mice after resuscitated hemorrhagic shock and tissue trauma (HS/T). A: (1) and (2) are the 2 methods used for gating ILC2. (1) ILC2 were gated using the surface markers CD25, CD127, CD90.2, suppression of tumorigenicity 2 (ST2), Sca-1, and CD117 after gating alive CD45^+^Lin^-^ cells. (2) ILC2 were gated using the surface markers CD25 and CD127 as well as the intracellular marker GATA3 after gating alive CD45^+^Lin^-^ cells. ILC2 gated by the 2 methods is shown in (3) and reveals that than 90% of CD45^+^Lin^-^CD25^+^ CD127^+^ CD90.2^+^ ST2^+^Sca-1^+^CD117^int^ cells were GATA3^+^ (see histogram: the right peak shows CD45^+^Lin^-^CD25^+^CD127^+^ CD90.2^+^ST2^+^Sca-1^+^CD117^int^ cells, which were also GATA3^+^, and the left peak shows isotype control). (4) More than 90% of CD45^+^Lin^-^CD25^+^ CD127^+^ GATA3^+^ cells were CD90.2^+^ST2^+^Sca-1^+^CD117^int^. B: ILC2 frequency (gated by CD45^+^Lin^-^CD25^+^CD127^+^CD90.2^+^ST2^+^Sca-1^+^CD117^int^) was gradually increased in the lung starting at 6 hours after HS/T and peaked at 24 hours (*n* = 5–8/group). C: ILC2 interleukin (IL) 5 (gated by CD45^+^Lin^-^CD25^+^CD127^+^GATA3^+^IL5^+^) increased in the lung after HS/T and was highest at 6 hours (*n* = 4–8/group). * *P* < 0.05. FSC-H, forward scatter-height.

ILC2 are a population of resident, TCR^-^, GATA3^+^ cells in the lungs that can respond to IL33 for the production of type 2 cytokines, including IL5 [25,26]. Although ILC2 have well-described roles in type 2 immune responses that evolve over a period of days to weeks [55,56], their role in acute inflammatory responses in the lung after trauma has not been characterized. Two gating methods were used to quantitate ILC2 in cells isolated from lungs of C57BL/6 mice (Fig 4A). First, ILC2 were gated using the surface markers CD25 and CD127 as well as CD90.2, ST2, Sca-1, and CD117 after gating for viable CD45^+^ Lin^-^ cells. Second, ILC2 were gated using the surface markers CD25 and CD127 combined with the intracellular marker GATA3 after gating for viable CD45^+^Lin^-^ cells. We found that more than 90% of CD45^+^Lin^-^CD25^+^CD127^+^CD90.2^+^ST2^+^Sca-1^+^CD117^int^ cells were GATA3^+^ and more than 90% of CD45^+^Lin^-^CD25^+^CD127^+^GATA3^+^ cells were CD90.2ST2^+^Sca-1^+^CD117^int^, which suggested the same population of ILC2 were gated through the 2 methods. Therefore, we used surface staining (CD45^+^Lin^-^CD25^+^CD127^+^CD90.2^+^ST2^+^Sca-1^+^CD117^int^) to establish changes in percentage of ILC2 or quantitate changes in IL5 expression by combining surface staining for CD45^+^Lin^-^CD25^+^CD127^+^ with intracellular staining for GATA3^+^ and IL5. We found the frequency of ILC2 increased in the lungs of C57BL/6 mice after HS/T. This increase could be detected at 6 hours and increased further at 24 hours (Fig 4B). The percentage of ILC2 that expressed intracellular IL5 also increased after HS/T but was highest at 6 hours (*P* < 0.05) (Fig 4C). Thus, ILC2 numbers and IL5 expression increased rapidly after severe trauma.

### ILC2 expression and activation as well as neutrophil IL5 up-regulation after HS/T are IL33 dependent

**Fig 5:**
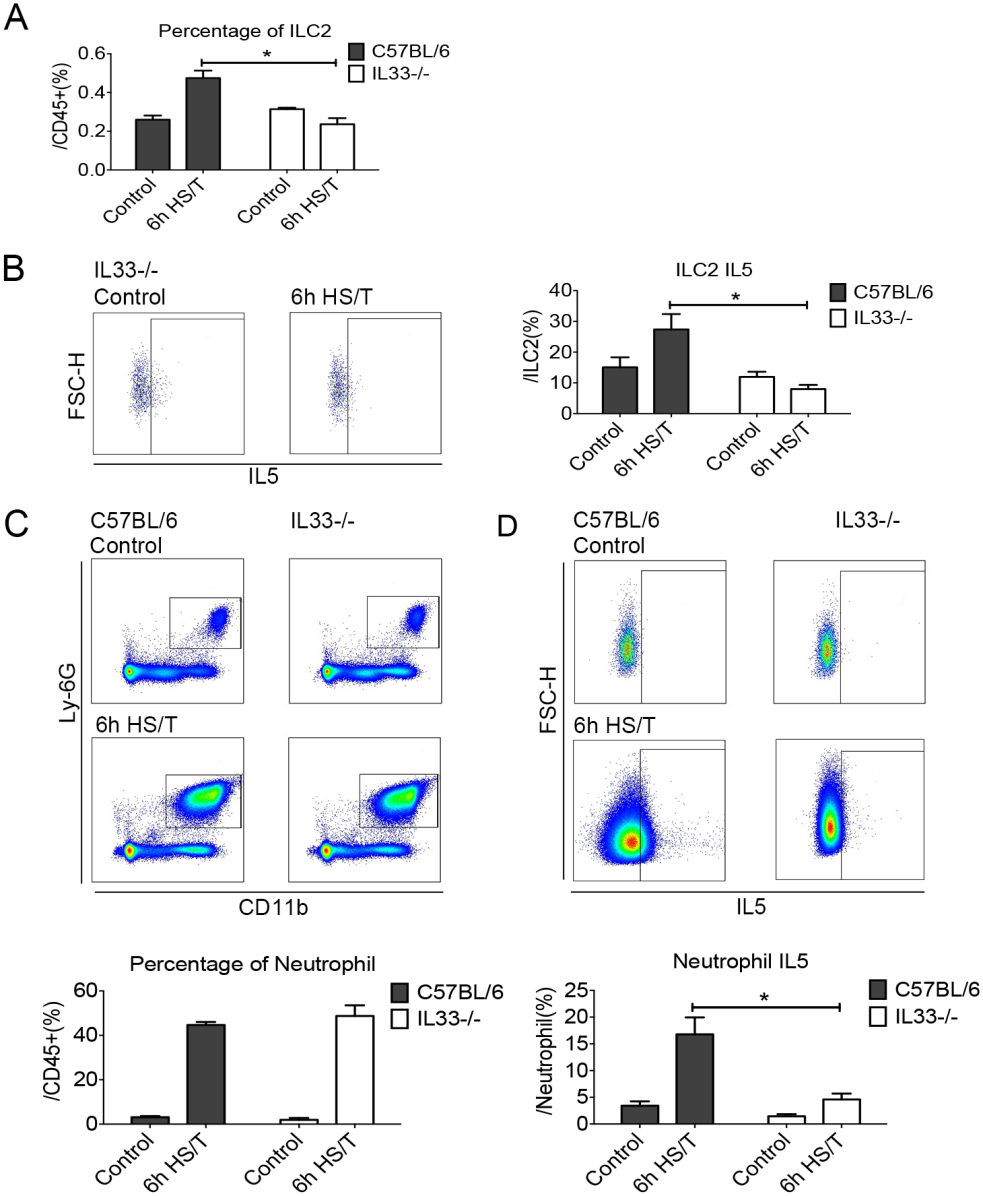
Effects of interleukin (IL) 33 deletion on group 2 innate lymphoid cells (ILC2) and neutrophil responses in lungs after resuscitated hemorrhagic shock and tissue trauma (HS/T). The increase of ILC2 frequency (A) and the ILC2 IL5 expression (B) at 6 hours after HS/T was inhibited in IL33^-/-^ mice (*n* = 5–8/group). C: The increase in neutrophil (Ly6G^+^CD11b^+^ gated from CD45^+^ cells) frequency after HS/T was not changed by IL33 deletion (*n* = 6–7/group). D: The increase of neutrophil IL5 expression (IL5^+^ gated from CD45^+^Ly6G^+^CD11b^+^ cells) at 6 hours after HS/T was inhibited in IL33^-/-^ mice, compared with the C57BL/6 WT mice (*n* = 5–7/group). * *P* < 0.05. FSC-H, forward scatter-height.

IL33 can activate ILC2 to produce type 2 cytokines in models of allergic asthma and atopic dermatitis [25–28]. We subjected IL33^-/-^ mice to HS/T and found that both the increase in ILC2 frequency and intracellular ILC2 IL5 expression at 6 hours after HS/T were dependent on IL33 (Fig 5A and 5B) (*P* < 0.05). Whereas the frequency of neutrophils in the lungs after HS/T was not influenced by IL33 deletion (Fig 5C), the up-regulation of neutrophil IL5 expression was dependent on IL33 (IL5^+^ cells gated from CD45^+^Ly6G^+^CD11b^+^) (*P* < 0.05) (Fig 5D). Immunohistochemistry of Ly6G also confirmed that neutrophils were up-regulated in the lungs of IL33^-/-^ mice at 6 hours after HS/T, like the up-regulation in the WT C57BL/6 mice (S4 Fig). This indicates that IL33 drives the increase in ILC2 numbers as well as the expression of IL5 in ILC2 and neutrophils but not neutrophil infiltration into the lungs after HS/T.

Two epithelial cytokines other than IL33, IL25, and thymic stromal lymphopoietin (TSLP) are known to activate ILC2 in the lung [22,24]. Although levels of both IL25 and TSLP increased in the plasma by 6 hours after HS/T (S5A and S6A Figs), pretreatment with neutralizing antibody to these cytokines had no impact on the increase in lung ILC2 or neutrophil IL5 expression at 6 hours (S5C and S6C Figs).

### The up-regulation of IL5 in neutrophils after HS/T requires ILC2

**Fig 6:**
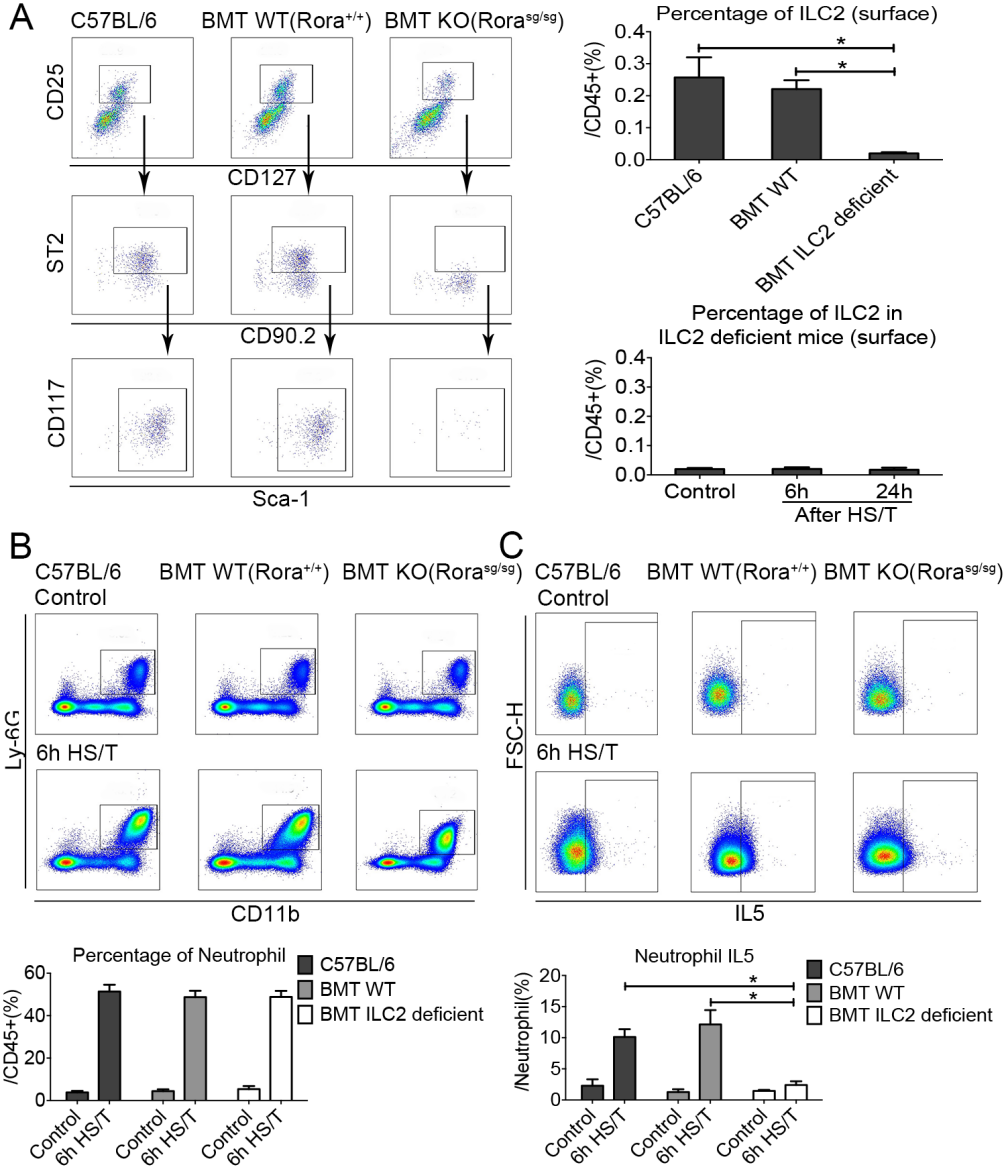
Effects of group 2 innate lymphoid cells (ILC2) deletion on lung frequency and interleukin (IL5) expression after resuscitated hemorrhagic shock and tissue trauma (HS/T). A: ILC2 nearly absent in ILC2-deficient Rora^sg/sg^ mice compared with the C57BL/6 or the bone marrow transplantation (BMT) wild-type (WT) (Rora^+/+^) mice, and no increase of ILC2 frequency was observed in ILC2-deficient mice after HS/T (*n* = 3–6/group). B: ILC2 deficiency had no impact on the increase in neutrophil frequency after HS/T (*n* = 5–8/group). C: The increase neutrophil IL5 expression after HS/T was inhibited in ILC2-deficient mice, compared with the C57BL/6 mice or the BMT WT mice (*n* = 5–6/group). * *P* < 0.05. FSC-H, forward scatter-height; KO, knockout.

ILC2-deficient mice were created by adoptive transfer of bone marrow from Rora^sg/sg^ mice into lethally irradiated B6.SJL-Ptprc Pepc mice as described by others [35]. We confirmed that ILC2 were depleted in the lungs of the bone marrow transplant ILC2-deficient (Rora^sg/sg^) mice at 12 weeks (*P* < 0.05), and no increase in ILC2 was observed in the lungs of the ILC2-deficient mice at 6 hours or 24 hours after HS/T (Fig 6A). Deletion of ILC2 did not change the frequency of neutrophils in the lung (Fig 6B) but ablated up-regulation of IL5 in neutrophils after HS/T (*P* < 0.05) (Fig 6C). Thus, up-regulation of IL5 by neutrophils is dependent on ILC2.

It is not feasible to isolate lung tissue for assessment of ILC2 populations in the lung after severe trauma in humans. Instead, we sought to establish evidence if ILC2 are engaged early after injury in humans. Circulating leukocytes were isolated from 4 severely injured blunt trauma patients upon admission and at 24 and 48 hours after admission. The numbers of ILC2 in the blood of the injured subjects were compared to the numbers in 3 healthy control subjects. The absolute counts of ILC2 were significantly lower in the trauma patients even at the time of the initial blood draw and remained lower for the first 48 hours after injury (S7 Fig). We hypothesize that the markedly lower ILC2 numbers in the circulation after severe trauma could reflect the migration of cells into the peripheral tissues.

### IL5 amplified neutrophil IL5 expression in lungs of C57BL/6 mice after HS/T

**Fig 7:**
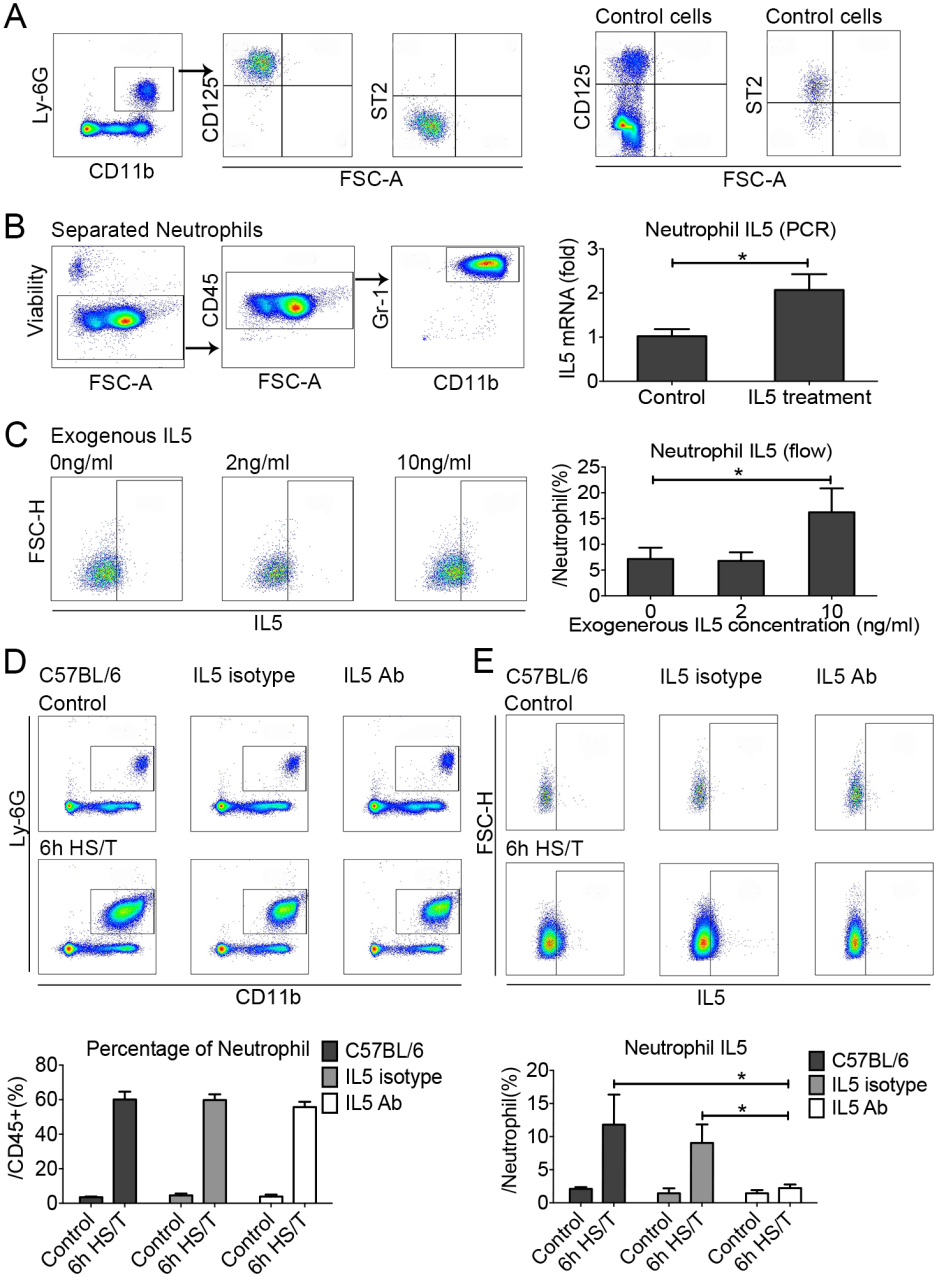
The effects of exogenous recombinant interleukin (IL) 5 or IL5 neutralization on neutrophil IL5 expression in the lungs of C57BL/6 mice after resuscitated hemorrhagic shock and tissue trauma (HS/T). A: Neutrophils in the lungs of C57BL/6 mice did not express suppression of tumorigenicity (ST2) but were positive for CD125 (IL5 receptor). The control cells for ST2 expression were gated from the CD45^+^Lin^-^CD25^+^CD127^+^, and the control cells for CD125 expression were gated from the CD45^+^ cells. B: Exposure of lung neutrophils isolated from HS/T mice to exogenous recombinant mouse IL5 (10 ng/ml) increased IL5 mRNA levels (*n* = 5/group, detected by PCR). C: Exposure of neutrophils isolated from the bone marrow to 10 ng/ml exogenous recombinant mouse IL5 induced their expression of IL5 (*n* = 3–7/group, detected by flow cytometry). D: IL5 neutralization did not change the frequency of neutrophils in the lungs after HS/T (*n* = 3–6/group). E: IL5 neutralization did inhibit the increase in lung neutrophil expression of IL5 after HS/T (*n* = 3–6/group). * *P* < 0.05. FSC-H, forward scatter-height.

The above data suggest that IL33 regulates neutrophil IL5 expression through signals released from ILC2 in response to IL33, and we hypothesized that IL5 from ILC2 regulates the expression of IL5 by neutrophils after HS/T. Up-regulation of IL5 in neutrophils after HS/T does not appear to be from direct IL33 stimulation, as neutrophils isolated from the lungs of C57BL/6 mice did not express ST2 (Fig 7A). Neutrophils, however, were shown to express the IL5 receptor, CD125 (Fig 7A). To confirm if IL5 could up-regulate IL5 expression by neutrophils and thus amplify the local type 2 response, neutrophils were isolated from the lungs of C57BL/6 mice 6 hours after HS/T using an anti-Ly6G microbead kit and restimulated with IL5. The isolated cell populations were over 99% neutrophils, with viability exceeding 99%. A subset of these neutrophils were then stimulated with exogenous recombinant mouse IL5 at 10 ng/ml for 45 minutes. IL5 mRNA levels were increased significantly by IL5 stimulation (*P* < 0.05) (Fig 7B). As additional evidence, neutrophils isolated from the bone marrow of C57BL/6 mice were stimulated with recombinant mouse IL5 (2 ng/ml or 10 ng/ml), and neutrophil intracellular IL5 was quantitated by flow cytometry. IL5 at 10 ng/ml was found to strongly induce the expression of IL5 by neutrophils (*P* < 0.05) (Fig 7C). More importantly, IL5 neutralizing antibody injected in vivo prior to HS/T did not alter neutrophil numbers (Fig 7D) but ablated increases in neutrophil IL5 expression (Fig 7E). In total, these data show that neutrophils increase their expression of IL5 in response to IL5, presumably derived from ILC2 stimulated with IL33.

### IL33 deletion, ILC2 deficiency, and IL5 neutralization prevent HS/T-induced lung injury

**Fig 8:**
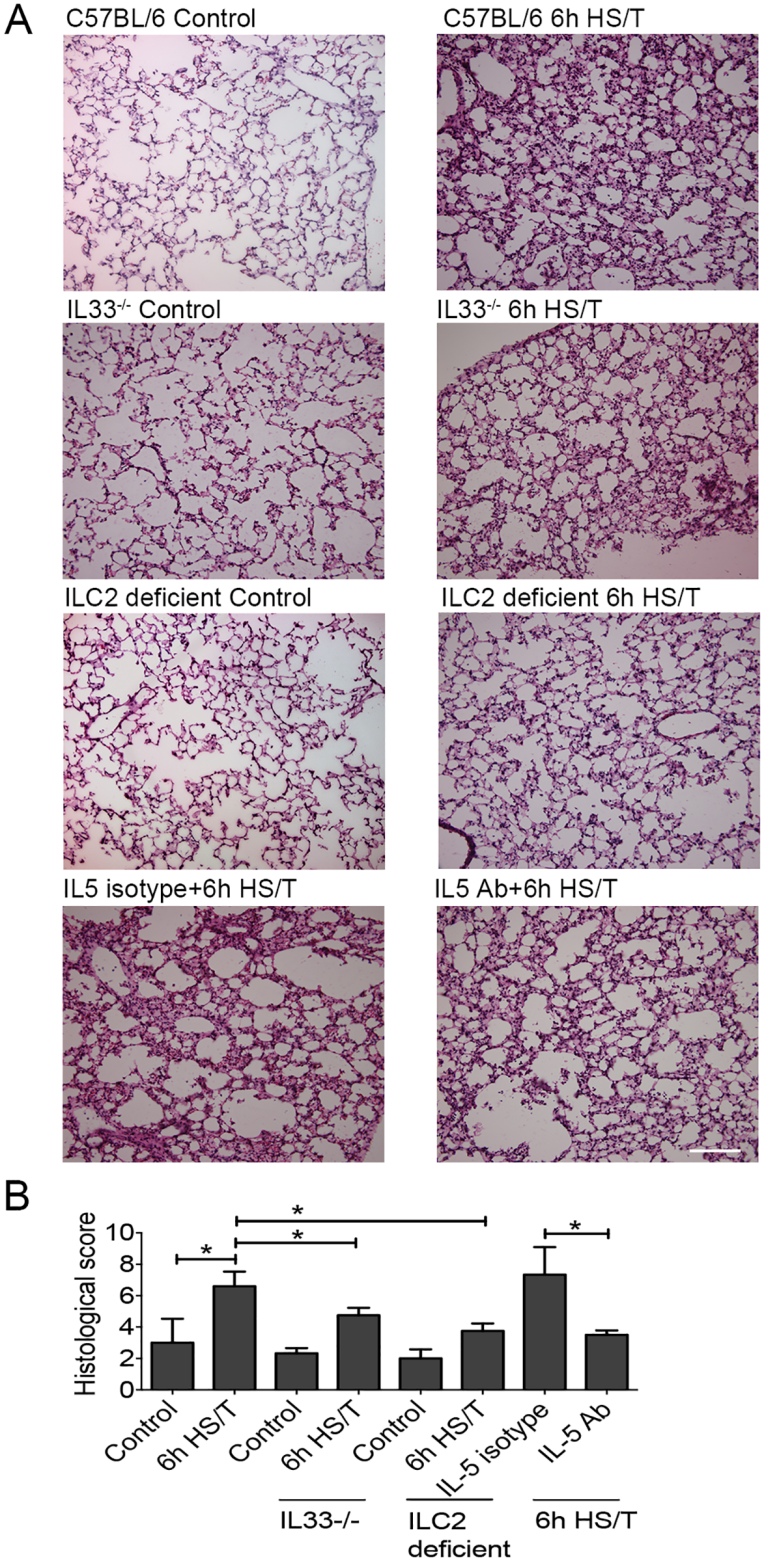
Effects of interleukin (IL) 33 deletion, ILC2 deficiency, and IL5 neutralization on lung injury after resuscitated hemorrhagic shock and tissue trauma (HS/T). A: Lung tissue sections stained with hematoxylin and eosin (H&E). Representative histologic sections are shown along with the average lung injury scores from control mice and at 6 hours after HS/T. IL33^-/-^ mice, mice with ILC2 deletion, and C57BL/6 WT mice pretreated with neutralizing anti-IL5 antibody are shown. 200× magnification; scale bar 100 μm. B: Lung injury scores were calculated on lung H&E sections (*n* = 3–5/group). * *P* < 0.05.

Severe trauma with hemorrhagic shock, as modeled here in mice, can lead to rapid lung injury and dysfunction [57,58]. To assess the impact of the IL33/ILC2/IL5/neutrophil pathway on lung changes 6 hours after HS/T in mice lacking IL33 or ILC2 or in which IL5 had been neutralized (Fig 8). Lung tissue slides were stained with H&E (Fig 8A), and lung injury was assessed by histological lung injury scoring (Fig 8B). The lung injury score assessed the degree of congestion and hemorrhage, the extent of edema and broadening of pulmonary alveolar wall, and the recruitment of inflammatory cells. As expected, HS/T led to a significant increase in lung injury scores in C57BL/6 mice (*P* < 0.05) (Fig 8B). In contrast, IL33 or ILC2 deletion, as well as IL5 neutralization, all significantly reduced lung injury scores at 6 hours following HS/T (Fig 8B). Thus, the rapid up-regulation of IL33 in the lungs induced by trauma with hemorrhagic shock can activate ILC2 within hours of injury and promote lung injury through ILC2 production of IL5.

## Discussion

Recent findings indicate that IL33-stimulated ILC2 are a major source of type 2 cytokines in the lung, where these resident innate lymphocytes potently regulate immune responses [24,26,59,60]. While severe trauma often results in acute lung injury, it was unclear if IL33 induced ILC2 cytokine production after systemic injury. It was also unknown if IL33-stimulated ILC2 in the lung shaped detrimental type 2 immune responses after trauma [7]. In determining if IL33 may drive potentially detrimental type 2 immune responses after severe trauma, we found that IL33 levels profoundly and rapidly increase in the blood after injury in humans. This increase was well ahead of subsequent increases in sST2, an endogenous IL33 antagonist [21]. The early increase in IL33 correlated with increased circulating type 2 cytokines, including IL5, and the development of NI and pulmonary dysfunction. Mechanistic studies using a rodent model of severe trauma that recapitulates the rapid escalation in systemic inflammation seen in injured humans revealed analogous findings of early increases in IL33 followed by delayed increases in sST2. The mouse model allowed us to demonstrate that IL33 drives ILC2 production of IL5 that then stimulates neutrophil IL5 in the lung after trauma with shock. Utilizing anti-IL5 antibodies, we established that IL5 was a dominant mediator of early, trauma-induced injury in the lungs. Thus, following a strategy of reverse translating observations made in human trauma into mechanistic studies in a preclinical model allowed us to develop evidence for a role of an IL33–ILC2–IL5–neutrophil axis in the early lung injury response induced by hemorrhagic shock with tissue trauma. These findings also extend the role of ILC2 to acute lung injury responses in noninfectious inflammation and show that IL33-activated ILC2 regulate the local responses of neutrophils recruited to the lung. Most importantly, the current studies identify IL33 and IL5 as potential therapeutic targets to prevent early pulmonary dysfunction in trauma patients.

The roles of type 2 immune responses depend on the tissue type and are important for antihelminth immunity, metabolic homeostasis, allergic inflammation, and reparative processes [25,61,62]. The hallmark of type 2 immunity is the production of type 2 cytokines, including IL4, IL5, IL9, and IL13. These cytokines can be produced by several cell types, such as Th2 lymphocytes, myeloid-derived suppressor cells (MDSCs), mast cells, and ILC2 to yield type 2 immunity [17,24,48,63,64].

After injury, those patients prone to complications, such as NI and MODS, have elevated type 2 cytokines in their circulation [7]. A polarization of CD4 lymphocyte subsets away from Th1 and towards Th2 is known to occur early after severe injury and has been proposed to contribute to impaired immune defenses [13,14,48,65]. Increased numbers of MDSCs also appear in trauma patients [66,67], and both Th2 cells and MDSCs can produce type 2 cytokines [15,63,64]. ILC2, while rare, are robust producers of type 2 cytokines, particularly IL5 [59,68]. Here, we show that ILC2 are lost from the circulation of injured humans, while circulating IL33, an alarmin and driver of type 2 immune responses, is rapidly increased. These observations led us to hypothesize that after trauma, IL33 released from cells is a central regulator of type 2 cytokines and their associated detrimental impacts. Thus, we reverse translated our clinical findings in a mouse model that combines hemorrhagic shock plus tissue trauma and show that this insult induces a rapid increase in IL33 expression in the lung and that IL33 activates IL5 production by ILC2. We also reveal that ILC2 IL5 secretion induces IL5 expression in infiltrating neutrophils. Thus, our present work elucidates a new role for ILC2 regulation of neutrophils in inflammation leading to lung injury responses after systemic injury.

IL33 is a member of the IL1 cytokine superfamily and functions as an alarmin when released from damaged or necrotic cells [21]. Membrane-bound ST2, also referred to as IL1-1R4, serves as the cellular receptor for IL33 on responsive cells such as CD4 T-cell subsets, including Th2 and some T regulatory cells, mast cells, and ILC2 [21]. We now show in both humans and mice that circulating IL33 levels are elevated early after injury. In patients that follow a complicated course (which includes pulmonary dysfunction and an increase in infection rates), the IL33 elevations are greater and persist over several days when compared to similarly injured patients with uncomplicated courses. In injured mice, we were able to show that IL33 is elevated in both the blood and lungs within 6 hours of injury. The soluble form of ST2 acts as an endogenous inhibitor of extracellular IL33 [44] and is elevated in many inflammatory and disease states [18,45,46]. The peak in sST2 levels in both injured humans and mice was observed several hours after the peak in IL33 in the plasma. These data suggest that there is a multihour window in which IL33 would be free to shape systemic immune responses after injury. A major difference between the clinical and experimental studies is the ability to set the 0 time point in the mice. The measurement of plasma mediators in humans can be delayed by prehospital events and the time needed for patient transportation. Therefore, the initial blood draw in humans could align with the 6-hour time point that we studied in the mice. Thus, the results from both the experimental and clinical measurements show concordance and seem to suggest that IL33 is released very early in the systemic response to injury and earlier than sST2. The correlation between IL33 (and not sST2) and the type 2 cytokines suggest that it is the early elevations in IL33 that set in motion and determine the extent of ILC2-mediated inflammation driven through IL5. This process may then be suppressed by the subsequent increase in extracellular sST2. ILC2-produced cytokines and reparative factors might have roles in the later phases of the clinical course of injured humans that are determined by the balance between IL33 and sST2. However, it was not feasible to investigate this aspect of the response in the mouse model.

Although IL33 can regulate type 2 responses through multiple cellular targets, we found that elevations of IL5 in the lung were entirely dependent on ILC2. In fact, the frequency of lymphocytes dropped in the lung after HS/T, and this included CD4^+^ cells. ILC2 are typically present in low numbers; however, the percentage of CD45^+^ cells that were ILC2 in the lung increased rapidly after systemic injury. Despite their low numbers, these noncytolytic, regulatory innate lymphoid cells are a potent source of type 2 cytokines [24,26,68]. In these studies we could only measure increases in IL5 in the lung after HS/T. Our experiments were not designed to give accurate whole-lung cell counts but instead yield a pure population of CD45+ cells. Therefore, it was not feasible to determine accurate tissue leukocyte cell counts in the lungs. However, ILC2 represent a low-abundance population of cells, and with the obvious influx of neutrophils into the lungs after HS/T, the parallel rapid increase in ILC2 frequency suggests that ILC2 numbers likely increase to a greater extent than estimated by the increase in frequency. Therefore, the measure of cell frequency (which we could measure accurately) allows us to conclude that lung ILC2 numbers increase rapidly after HS/T.

Other epithelial cytokines, including IL25 and TSLP, also regulate ILC2 function. We found that IL25 and TSLP were elevated in the plasma of mice subjected to HS/T, and both were present at baseline in the lungs. Neutralizing antibodies delivered to either IL25 or TSLP did not alter the production of IL5 by ILC2 or neutrophils after HS/T (S5 and S6 Figs). Recent findings suggest that 2 separate ILC2 populations can be present in the lungs of mice. These include a natural population (nILC2) that are responsive to IL33 for the production of IL5 and an inducible or inflammatory population (iILC2) that are ST2^-^ and emerge in response to IL25 [69]. However, the appearance of IL25-responsive iILC2 requires 2–3 days of in vivo IL25 exposure. The rapid, IL33-dependent up-regulation of IL5 in ILC2 after HS/T in these otherwise naïve mice is most likely due to the activation of nILC2.

Other than ILC2, we found that CXCR2^+^ neutrophils were the major source of IL5 in the lung after HS/T. Neutrophils are ST2-negative, which led us to show that neutrophils express the IL5 receptor and can respond to IL5 to produce their own IL5. Thus, we conclude that one of the important effects of IL33 in the lung after systemic shock and injury is the rapid release of IL5 by ILC2, which leads to more IL5 production by neutrophils that have been attracted to the lung as the result of increased CXCR2 expression. IL5 can stimulate neutrophil H_2_O_2_ release [70,71], which could account for the IL33-dependent lung injury that appears within hours of severe HS/T. Here, we show that neutralization of IL5, ILC2 deletion, and IL33 deletion all prevent the lung injury that appears within the first 6 hours after HS/T.

We found that CXCR2^+^ neutrophils expressed more IL5 than CXCR2^-^ neutrophils after HS/T. Others have shown that CXCR2^+^ is important for neutrophils to migrate to sites of inflammation [72]. Deletion of CXCR2 leads to less inflammation and lower type 2 cytokines (IL4 and IL5) in the lungs during allergic airway inflammation [73]. IL33-induced CXCR2 up-regulation is required for neutrophil activation in a mouse model of asthma mediated by immunoglobulin E [74]. We did not find a role for IL33 in regulating the increase in neutrophil numbers in the lung after HS/T. Therefore, other pathways likely contribute to neutrophil influx in the lung after HS/T.

The strengths of this study include conclusive data on the correlation of IL33 with type 2 cytokines in an adequately powered study of injured humans as well as the definitive mechanistic studies in a mouse polytrauma model that show IL33 activates ILC2 in the lungs, leading to neutrophil IL5 production. However, there are important limitations. The observations in humans are only correlations. The degree to which the mechanistic studies in mice translate back to the human condition is uncertain. Because there is a preponderance of male severely injured patients, we chose to include only male mice in this initial study. Future studies should include a comparison between males and females. Finally, we did not show whether the histopathologic changes observed in the lungs of mice correlate with changes in pulmonary function.

In summary, our studies demonstrate how reverse translation of human observations into mechanistic experimental models can be used to gain insights into trauma-induced immune dysfunction. This strategy allowed us to identify an unsuspected role for IL33-mediated activation of ILC2 in the initial inflammatory response in the lung after systemic injury. ILC2 regulate neutrophil function through IL5, which promotes IL5 expression in neutrophils and drives lung inflammatory changes. As lung dysfunction is a common and serious problem in patients experiencing hemorrhagic shock and tissue trauma, the findings may have identified a novel contributing pathway to this pathologic process that could be targeted through blocking IL33, IL5, or their receptors.

## Supporting information

S1 Fig Spearman correlation of injury severity score with admission IL33 levels for the first blood draw within the first 12 hours postinjury. *N* = 335. Correlation coefficient: –0.13, *P* = 0.015.
(TIF)

S2 Fig Time course analysis of plasma IL33 in blunt trauma males (*n* = 330) compared to females (*n* = 142). There were no differences in the average levels between female and male subjects.
(TIF)

S3 Fig Changes in eosinophil frequency in the lung of mice after HS/T (*n* = 3–6/group). Eosinophils in the lungs were evaluated by gating on CD45^+^Siglec-F^+^ cells; no increase is shown after HS/T.
(TIF)

S4 Fig Change in the Ly6G staining in the lung of IL33 KO mice after HS/T. Immunohistochemistry of Ly6G also confirmed that neutrophils were up-regulated in the lungs of IL33^-/-^ mice at 6 hours after HS/T, like the up-regulation in the WT C57BL/6 mice. Red, neutrophil; blue, nuclei; green, actin. 20 x 2z magnification, scale bar 50 μm.
(TIF)

S5 Fig Effects of IL25 on neutrophil IL5 as well as ILC2 IL5 expression in the lungs of mice after HS/T. A: The IL25 level in plasma was increased after HS/T, especially at 6 hours (*n* = 4–6/group, by ELISA). B: IL25 in the lungs maintained at a constant level after different time points after HS/T (*n* = 2–6/group, by ELISA). C: IL25 neutralization antibody showed no obvious effects on the increased neutrophil IL5 or ILC2 IL5 expression after HS/T (*n* = 2–5/group). * *P* < 0.05.
(TIF)

S6 Fig Effects of TSLP on neutrophil IL5 as well as ILC2 IL5 expression in the lungs of mice after HS/T. A: TSLP increased gradually after HS/T in plasma (*n* = 4–8/group, by ELISA). B: TSLP level did not show obvious change in the lungs after HS/T. C: Neutralizing antibody delivered to TSLP did not show obvious effects on the increased neutrophil IL5 or ILC2 IL5 expression after HS/T (*n* = 2–5/group). * *P* < 0.05.
(TIF)

S7 Fig Changes in ILC2 numbers in the peripheral blood of trauma patients. ILC2 in peripheral blood leukocytes (PBL) were evaluated by gating on CD3 negative and lineage-negative cells, followed by gating on CD127^+^, CRTH2^+^. The absolute counts of ILC2 were significantly lower in the trauma patients even at the time of the initial blood draw and remained lower for the first 48 hours after injury. Controls, *n* = 3; patients, *n* = 5. Cell number x 10^3^/ml of blood is depicted. * *P* < 0.05.
(TIF)

S1 Table Overall demographics and clinical outcomes of the stringently matched infection group (*n* = 44) and the group with no infection (*n* = 44). Values are expressed as mean ± SEM. Mann–Whitney U-Test and Fisher’s exact test were used as appropriate with statistical significance set at *P* < 0.05.
(DOCX)

S1 Text The STROBE guidelines checklist for the human cohort data in this manuscript. (DOC)

S2 Text The prospective analysis plan for this manuscript. The prospective analysis plan was submitted as part of our IRB application on November 2015.
(DOCX)

S3 Text The ARRIVE Guidelines Checklist for the animal data in this manuscript. (DOCX)

## Abbreviations

ARDS: acute respiratory distress syndrome
BMT: bone marrow transplantation
BSA: bovine serum albumin
CC: correlation coefficient
DAMP: damage-associated molecular pattern
FSC-H: forward scatter-height
H&E: hematoxylin and eosin
HS: hemorrhagic shock
HS/T: resuscitated hemorrhagic shock and tissue trauma
HV: healthy volunteer
ICU: intensive care unit
iILC2: inflammatory population of group 2 innate lymphoid cells
IL: interleukin
ILC2: group 2 innate lymphoid cells
ISS: injury severity score
KO: knockout
mAb: monoclonal antibodies
MAP: mean arterial pressure
MDSC: myeloid-derived suppressor cell
MODS: multiple organ dysfunction syndrome
MOF: multiple organ failure
NI: nosocomial infection
nILC2: natural population of group 2 innate lymphoid cells
NK: natural killer
PBB: PBS containing 0.5% BSA
PF: pseudofracture
sST2: soluble ST2
ST2: suppression of tumorigenicity
TSLP: thymic stromal lymphopoietin
WT: wild type
