# Supplementary figures and images for: IL33-mediated ILC2 activation and neutrophil IL5 production in the lung response after severe trauma: A reverse translation study from a human cohort to a mouse trauma model
Source: PLoS Med. 2017 Jul 25;14(7):e1002365. doi: 10.1371/journal.pmed.1002365 (PMC5526517; doi:10.1371/journal.pmed.1002365)

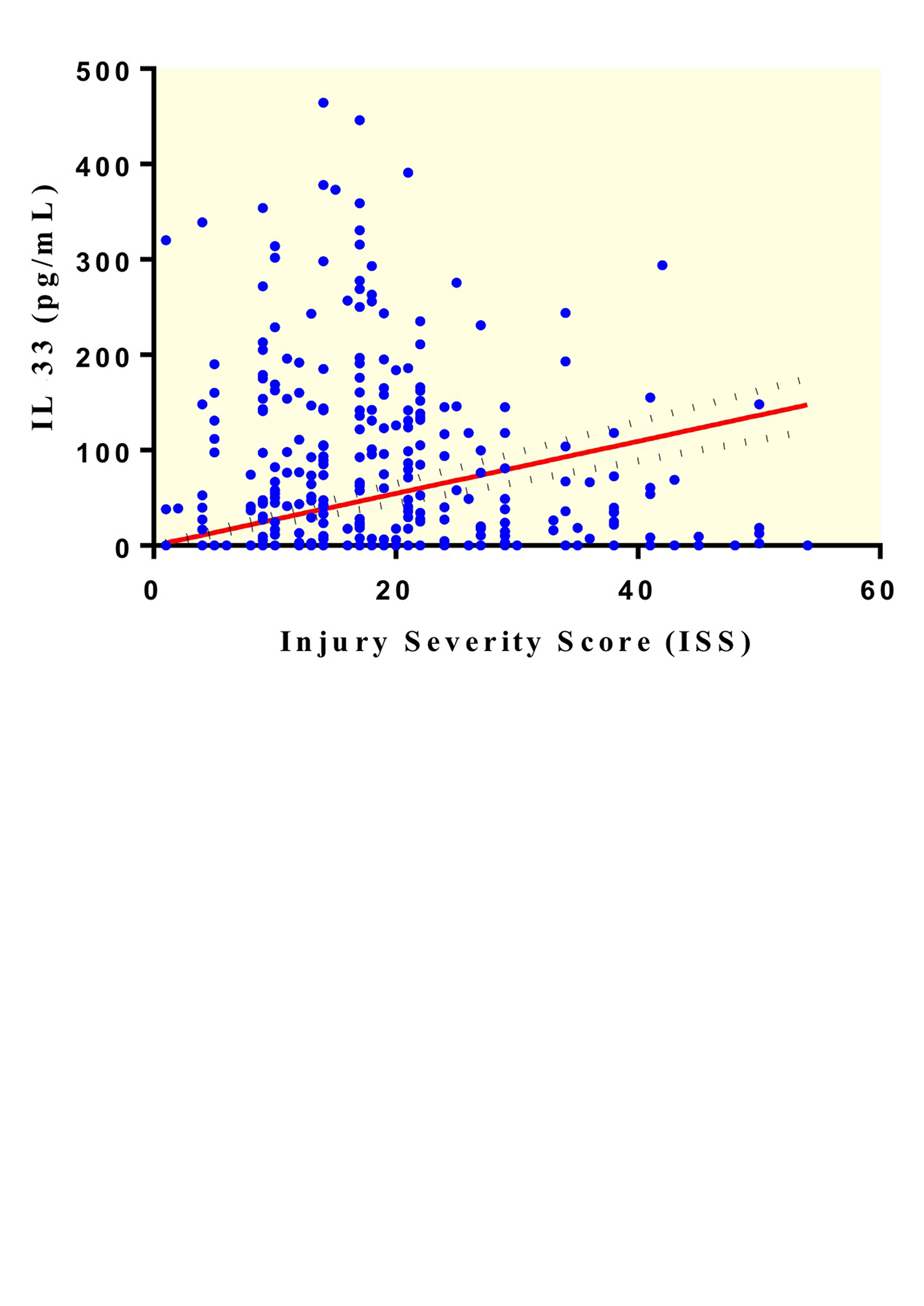

Supplement: S1 Fig — N = 335. Correlation coefficient: –0.13, P = 0.015. (TIF) [file pmed.1002365.s001.tif]

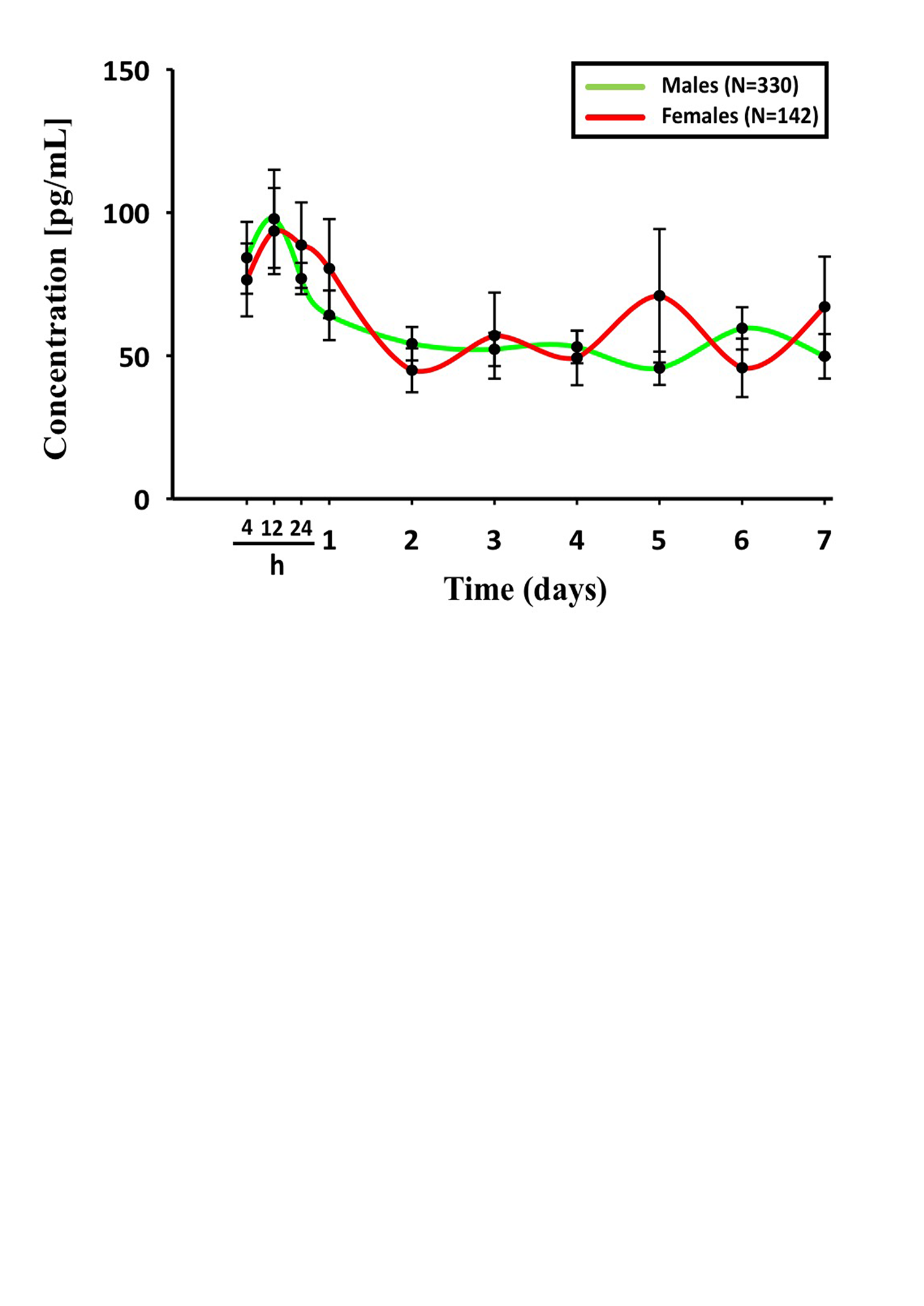

Supplement: S2 Fig — There were no differences in the average levels between female and male subjects. (TIF) [file pmed.1002365.s002.tif]

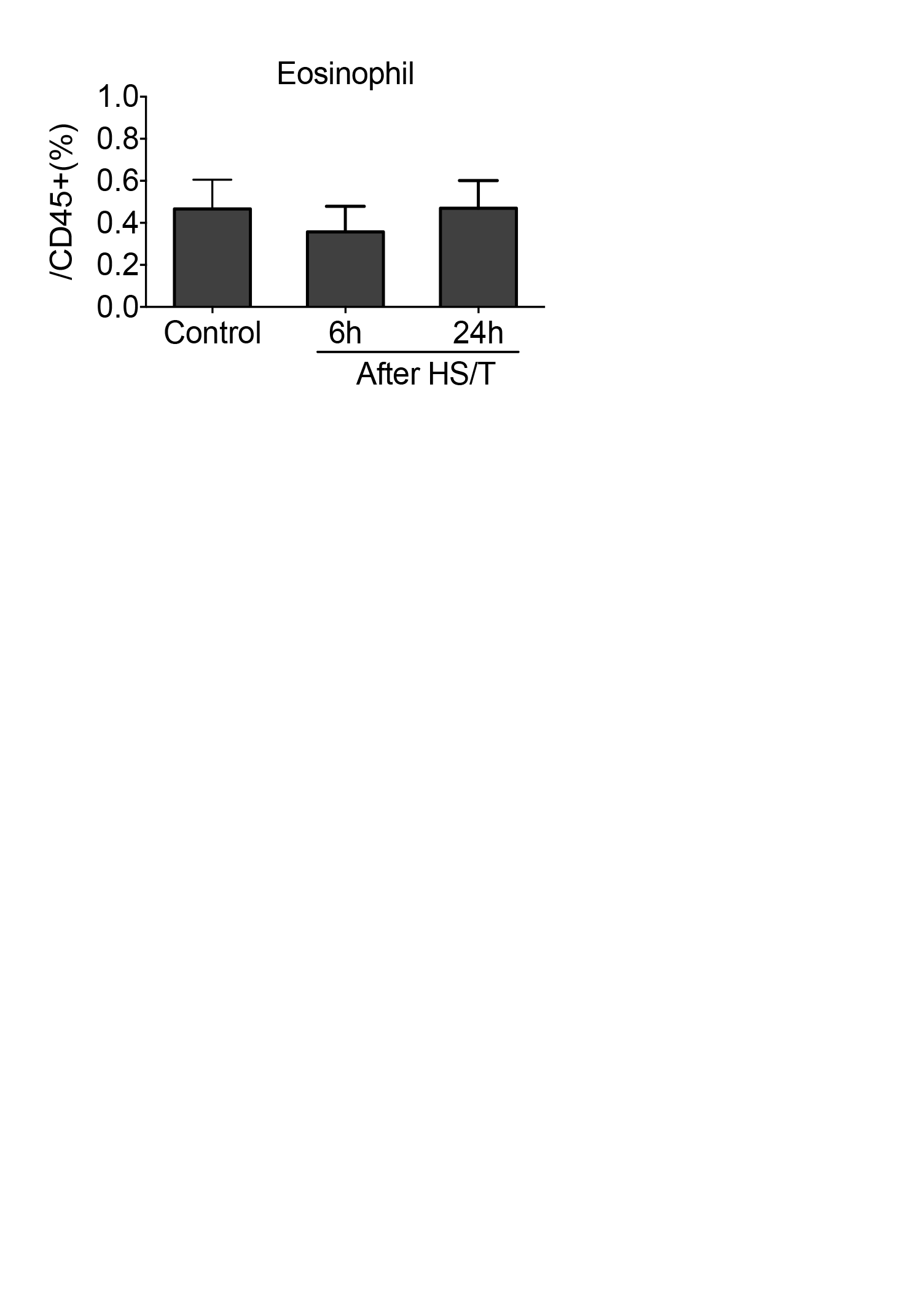

Supplement: S3 Fig — Eosinophils in the lungs were evaluated by gating on CD45+Siglec-F+ cells; no increase is shown after HS/T. (TIF) [file pmed.1002365.s003.tif]

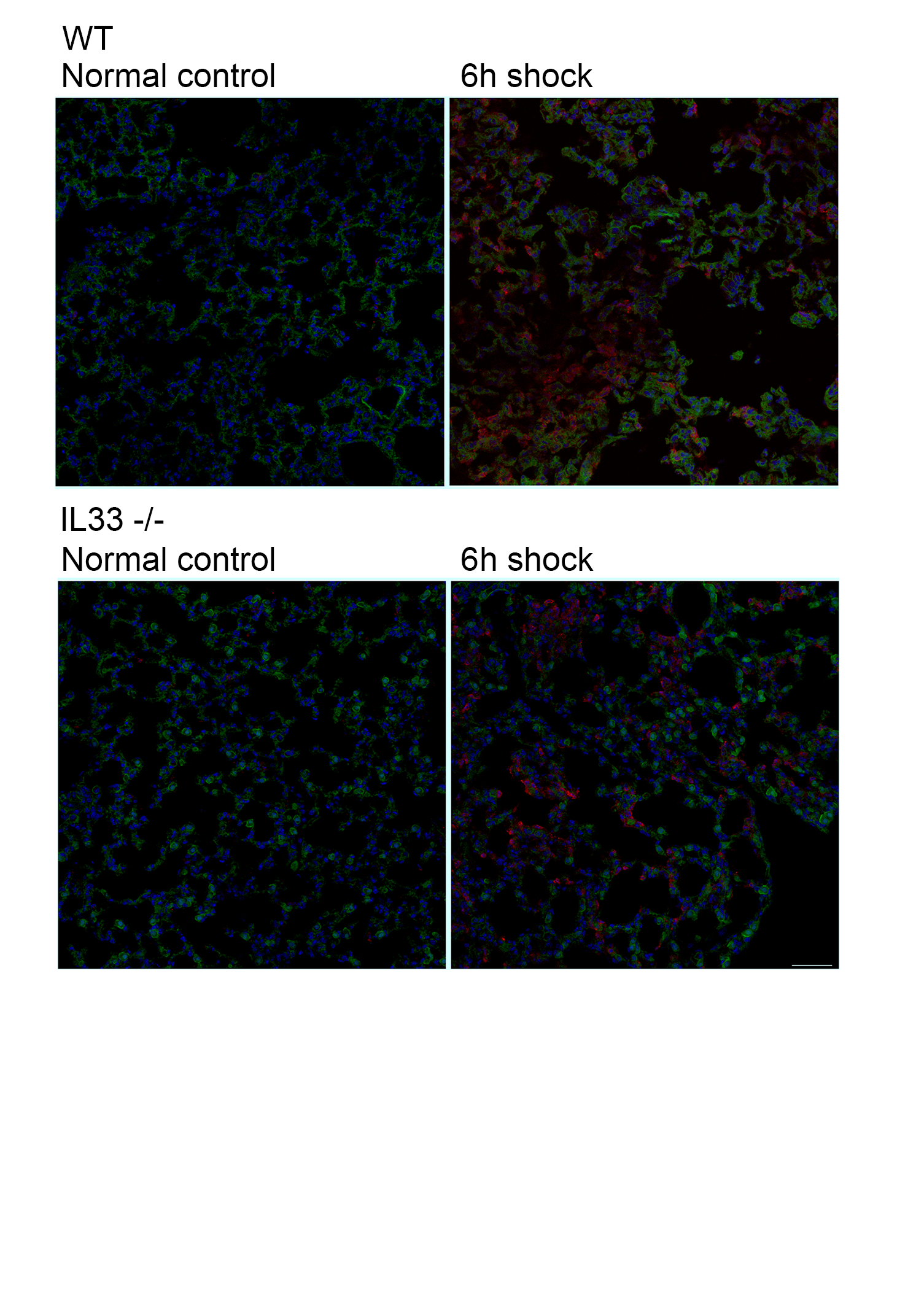

Supplement: S4 Fig — Immunohistochemistry of Ly6G also confirmed that neutrophils were up-regulated in the lungs of IL33-/- mice at 6 hours after HS/T, like the up-regulation in the WT C57BL/6 mice. Red, neutrophil; blue, nuclei; green, actin. 20 x 2z magnification, scale bar 50 μm. (TIF) [file pmed.1002365.s004.tif]

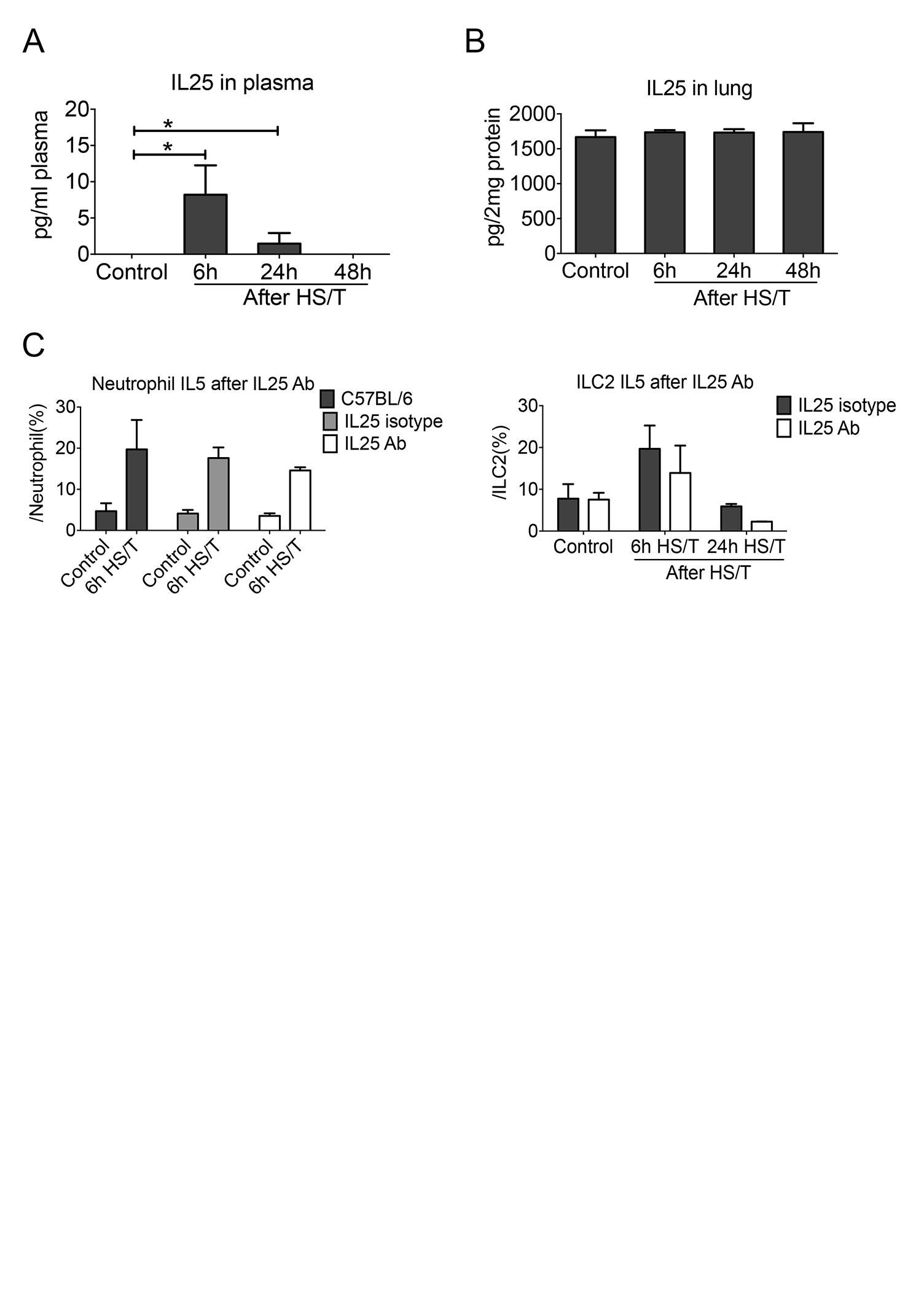

Supplement: S5 Fig — A: The IL25 level in plasma was increased after HS/T, especially at 6 hours (n = 4–6/group, by ELISA). B: IL25 in the lungs maintained at a constant level after different time points after HS/T (n = 2–6/group, by ELISA). C: IL25 neutralization antibody showed no obvious effects on the increased neutrophil IL5 or ILC2 IL5 expression after HS/T (n = 2–5/group). * P < 0.05. (TIF) [file pmed.1002365.s005.tif]

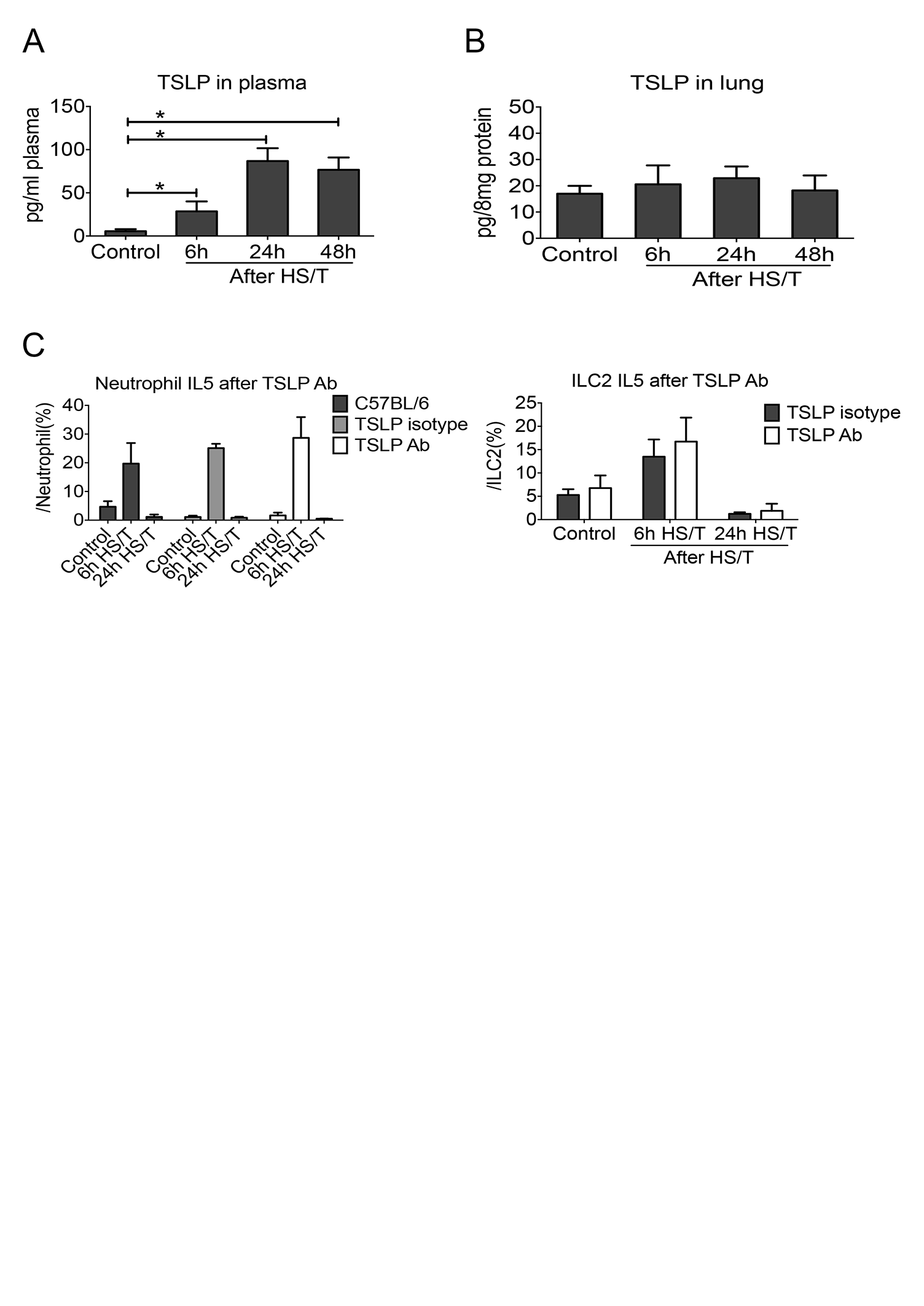

Supplement: S6 Fig — A: TSLP increased gradually after HS/T in plasma (n = 4–8/group, by ELISA). B: TSLP level did not show obvious change in the lungs after HS/T. C: Neutralizing antibody delivered to TSLP did not show obvious effects on the increased neutrophil IL5 or ILC2 IL5 expression after HS/T (n = 2–5/group). * P < 0.05. (TIF) [file pmed.1002365.s006.tif]

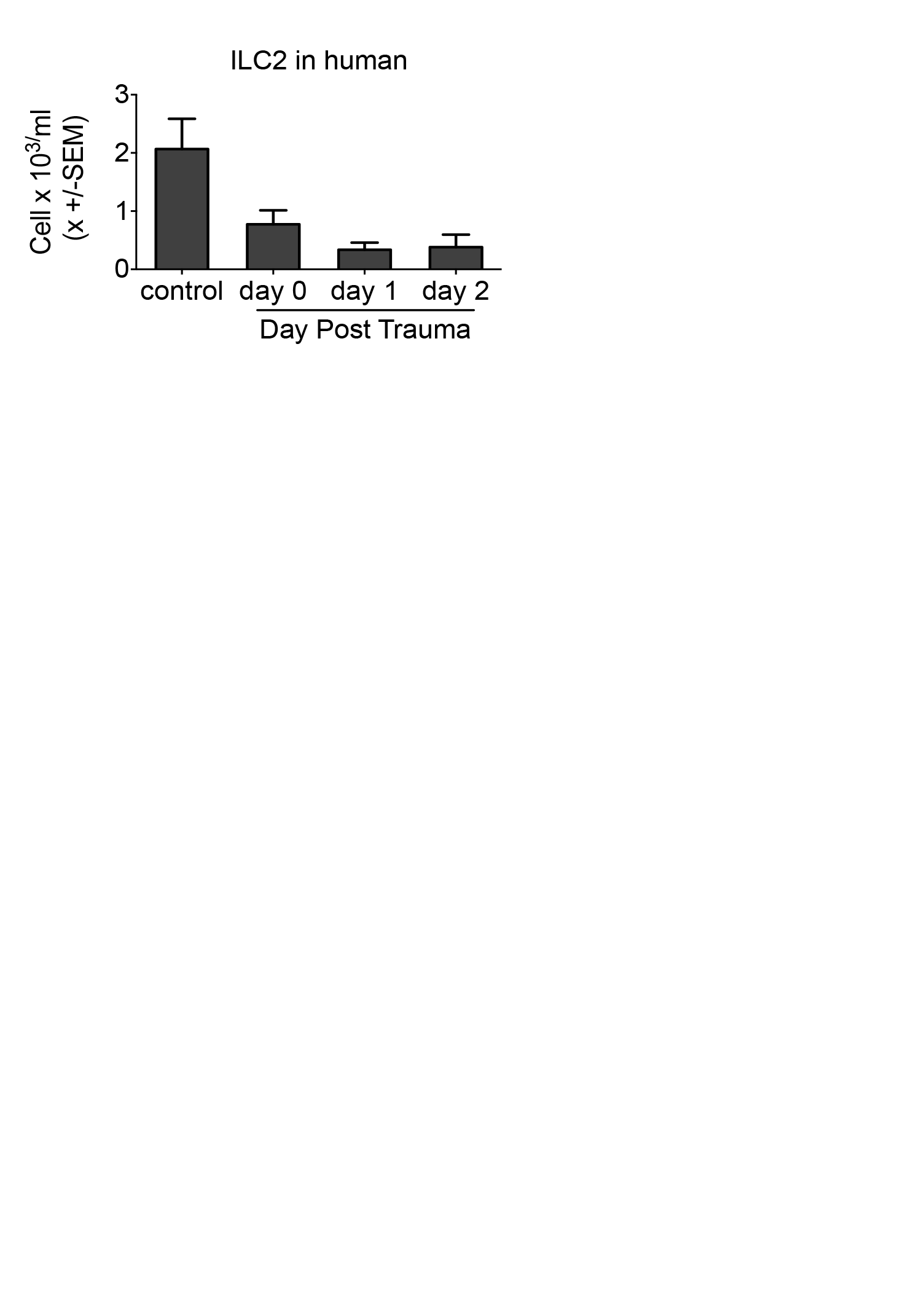

Supplement: S7 Fig — ILC2 in peripheral blood leukocytes (PBL) were evaluated by gating on CD3 negative and lineage-negative cells, followed by gating on CD127+, CRTH2+. The absolute counts of ILC2 were significantly lower in the trauma patients even at the time of the initial blood draw and remained lower for the first 48 hours after injury. Controls, n = 3; patients, n = 5. Cell number x 103/ml of blood is depicted. * P < 0.05. (TIF) [file pmed.1002365.s007.tif]
