# Supplementary material for: IL33-mediated ILC2 activation and neutrophil IL5 production in the lung response after severe trauma: A reverse translation study from a human cohort to a mouse trauma model
Source: PLoS Med. 2017 Jul 25;14(7):e1002365. doi: 10.1371/journal.pmed.1002365 (PMC5526517; doi:10.1371/journal.pmed.1002365)
Supplement: S1 Table — Values are expressed as mean ± SEM. Mann–Whitney U-Test and Fisher’s exact test were used as appropriate with statistical significance set at P < 0.05. (DOCX) [file pmed.1002365.s008.docx]

|  | **Infection**  **N = 44** | **No-Infection**  **N = 44** | ***P* value** |
| --- | --- | --- | --- |
| **Demographics** | | | |
| Age, yr | 48 ± 3 | 47 ± 2.3 | 0.835 |
| Sex, female/male | F=17 M=27 | F=17 M=27 | 1 |
| Injury Severity Score (ISS) | 26.3 ± 1.7 | 26 ± 0.9 | 0.993 |
| **Outcomes** | | | |
| Intensive Care Unit length of stay, days | 15 ± 1.5 | 4.5 ± 0.7 | 0.001 |
| Mechanical ventilation, days | 9.8 ± 1.4 | 1.8 ± 0.7 | 0.001 |
| Hospital length of stay, days | 23 ± 1.7 | 10.8 ± 1.2 | 0.001 |

**S1 Table.** **Overall demographics and clinical outcomes of the stringently matched infection (n=44) and no-infection (n=44) groups.** Values are expressed as mean ± SEM. Mann-Whitney U-Test and Fisher’s exact test were used as appropriate with statistical significance set at *P* < 0.05.
