# Supplementary material for: IL33-mediated ILC2 activation and neutrophil IL5 production in the lung response after severe trauma: A reverse translation study from a human cohort to a mouse trauma model
Source: PLoS Med. 2017 Jul 25;14(7):e1002365. doi: 10.1371/journal.pmed.1002365 (PMC5526517; doi:10.1371/journal.pmed.1002365)
Supplement: S1 Text — (DOC) [file pmed.1002365.s009.doc]

STROBE Statement—Checklist of items that should be included in reports of ***cohort studies***

|  | Item No | Recommendation |
| --- | --- | --- |
| **Title and abstract** | 1 | (*a*) Indicate the study’s design with a commonly used term in the title or the abstract  The study design was referred to in the title: “*a reverse translation study from a human cohort to a mouse trauma model*”. In the abstract, methods and findings section: “*Based on the observations in humans, mechanistic experiments were designed in a mouse model of resuscitated hemorrhagic shock and tissue trauma (HS/T*)”. |
|  |  | (*b*) Provide in the abstract an informative and balanced summary of what was done and what was found  Included in the abstract, conclusions section: “*These results suggest that IL-33 may initiate early detrimental type 2 immune responses after trauma through ILC2 regulation of neutrophil IL-5 production. This IL-33/ILC2/IL-5/neutrophil axis defines a novel regulatory role for ILC2 in acute lung injury that could be targeted in trauma patients prone to early lung dysfunction*”. |
| Introduction | | |
| Background/rationale | 2 | Explain the scientific background and rationale for the investigation being reported  We proposed the utility of “*reverse translation of novel observations made in humans in well-characterized experimental models has been proposed as an efficient way to gain insights into the mechanisms of immune dysfunction after severe injury*” Introduction, paragraph 1. Based on prior observations “*We have recently provided evidence that trauma patients that go on to develop nosocomial infection also exhibit higher levels of type 2 cytokines, including IL-4, IL-5, and IL-13 within 24 hours after injury”* Introduction, paragraph 2.  “*IL-33, a member of the IL-1 cytokine super-family, is constitutively expressed in the epithelium of both mice and humans and acts as an alarmin after tissue injury by activating immune cells through its cognate receptor, ST2. Group 2 innate lymphoid cells (ILC2) are a recently described subset of linage- ST2+, GATA3-dependent, lymphocytes found in the subepithelial space in the lungs and other barrier organs, as well as adipose tissue. ILC2 are non-cytolytic, but can be activated to produce the type 2 cytokines IL-5 and IL-13 in response to IL-33 and other signals from the adjacent epithelium*.” Introduction, paragraph 3. |
| Objectives | 3 | State specific objectives, including any prespecified hypotheses  “*the role of ILC2, stimulated by the release of IL-33 post-injury, to support potentially detrimental type 2 responses is not yet fully elucidated*. *Accordingly, we tested the hypothesis that IL-33 drives an early type 2 immune response in the lung through the activation of resident ILC2*.” Introduction, paragraph 4. |
| Methods | | |
| Study design | 4 | Present key elements of study design early in the paper  The study design including human blunt trauma enrolment, data collection, and blood sampling were introduced early in the Methods section, trauma patient enrollment and data collection, paragraph 3. |
| Setting | 5 | Describe the setting, locations, and relevant dates, including periods of recruitment, exposure, follow-up, and data collection  All these points were included under the section “Trauma patient enrollment and data collection”. Methods, paragraph 3. |
| Participants | 6 | (*a*) Give the eligibility criteria, and the sources and methods of case ascertainment and control selection. Give the rationale for the choice of cases and controls  Eligibility criteria included “*victims of blunt force trauma, at least 18 years of age and admitted to the ICU.”* Methods, paragraph 3. For baseline control biomarker levels, 12 healthy volunteers with no recent illness or infection in the last two weeks-no recent hospitalization or trauma in the last month prior to recruitment. Results, paragraph 1. The matched case-control groups’ (i.e. the 44 nosocomial infection group and the 44 no-infection group) were recently reported in the Annals of Surgery (reference # 7). Results, paragraph 3. |
|  |  | (*b*) For matched studies, give matching criteria and the number of controls per case  The matched groups were selected “*by comparing cohorts with or without NI (n=44 each) matched for injury severity (based on ISS), age, and sex to exclude potential confounders based on differences in these variables*.” Results, paragraph 3. |
| Variables | 7 | Clearly define all outcomes, exposures, predictors, potential confounders, and effect modifiers. Give diagnostic criteria, if applicable  Primary outcomes included: “*Nosocomial infection was diagnosed on day 6 on average and we have previously reported that patients in the NI cohort also experience significantly higher incidences of other adverse in-hospital outcomes including prolonged respiratory failure, higher degree of MODS, and longer ICU and hospital stay*”. Results, paragraph 3. |
| Data sources/ measurement | 8* | For each variable of interest, give sources of data and details of methods of assessment (measurement). Describe comparability of assessment methods if there is more than one group  The injury severity score, age and gender data “*were recorded in the database via direct interface with the electronic medical record*.” Methods, paragraph 3. |
| Bias | 9 | Describe any efforts to address potential sources of bias  All human patient data was de-identified during enrollment, data acquisition, and data analysis. Data collection from the electronic medical record and analyses were performed by two different post-doctoral fellows. |
| Study size | 10 | Explain how the study size was arrived at  Patient enrollment covered 8 year period based on an inclusion and exclusion criteria. During this period 472 blunt trauma survivors were recruited for the study. Methods, paragraph 3. |
| Quantitative variables | 11 | Explain how quantitative variables were handled in the analyses. If applicable, describe which groupings were chosen and why  Not applicable. |
| Statistical methods | 12 | (*a*) Describe all statistical methods, including those used to control for confounding  Statistical methods included “*unpaired two-tailed Student’s t-test or Fisher’s exact test, as appropriate.*” Methods, paragraph 11. |
|  |  | (*b*) Describe any methods used to examine subgroups and interactions  “*Group-time interaction of plasma inflammatory mediators’ levels were determined by Two-Way Analysis of Variance (ANOVA).”* Methods, paragraph 11. |
|  |  | (*c*) Explain how missing data were addressed  All patients’ demographics and clinical data were complete. In six patients, biomarker data at different time points were missing and these were not included biomarker analysis. |
|  |  | (*d*) If applicable, explain how matching of cases and controls was addressed  Using IBM SPSS case-control matching algorithm controlling for age, sex, and ISS. Results, paragraph 3, Ref. #7. |
|  |  | (*e*) Describe any sensitivity analyses  Not applicable. |
| Results | | |
| Participants | 13* | (a) Report numbers of individuals at each stage of study—eg numbers potentially eligible, examined for eligibility, confirmed eligible, included in the study, completing follow-up, and analysed.  Recently reported. See Ref. #7 |
|  |  | (b) Give reasons for non-participation at each stage  Not applicable |
|  |  | (c) Consider use of a flow diagram  Recently reported. See Ref. #7 |
| Descriptive data | 14* | (a) Give characteristics of study participants (eg demographic, clinical, social) and information on exposures and potential confounders  Patient demographics and clinical outcomes are reported in supplemental Table S8. Also see Ref. #7. |
|  |  | (b) Indicate number of participants with missing data for each variable of interest  All data was complete. |
| Outcome data | 15* | Report numbers in each exposure category, or summary measures of exposure  Not applicable. |
| Main results | 16 | (*a*) Give unadjusted estimates and, if applicable, confounder-adjusted estimates and their precision (eg, 95% confidence interval). Make clear which confounders were adjusted for and why they were included  Not applicable. |
|  |  | (*b*) Report category boundaries when continuous variables were categorized  Not applicable |
|  |  | (*c*) If relevant, consider translating estimates of relative risk into absolute risk for a meaningful time period  Not applicable |

| Other analyses | 17 | Report other analyses done—eg analyses of subgroups and interactions, and sensitivity analyses  Not applicable |
| --- | --- | --- |
| Discussion | | |
| Key results | 18 | Summarise key results with reference to study objectives  *“In determining if IL-33 may drive potentially detrimental type 2 immune responses after severe trauma, we found that IL-33 levels profoundly and rapidly increase in the blood after injury in humans. This increase was well ahead of subsequent increases in sST2, an endogenous IL-33 antagonist. The early increase in IL-33 correlated with increased circulating type 2 cytokines, including IL-5, and the development of NI and pulmonary dysfunction. Mechanistic studies using a rodent model of severe trauma that recapitulates the rapid escalation in systemic inflammation seen in injured humans, revealed analogous findings of early increases in IL-33 followed by delayed increases in sST2. The mouse model allowed us to demonstrate that IL-33 drives ILC2 production of IL5 which then stimulates neutrophil IL-5 in the lung after trauma with shock. Utilizing anti-IL-5 antibodies, we established that IL-5 was a dominant mediator of early, trauma-induced injury in the lungs. Thus, following a strategy of reverse translating observations made in human trauma into mechanistic studies in a pre-clinical model allowed us develop evidence for a role of an IL-33/ILC2/IL-5/Neutrophil axis in the early lung injury response induced by hemorrhagic shock with tissue trauma. These findings also extend the role of ILC2 to acute lung injury responses in non-infectious inflammation and show that IL-33-activated ILC2 regulate the local responses of neutrophils recruited to the lung. Most importantly, the current studies identify IL-33 and IL-5 as potential therapeutic targets to prevent early pulmonary dysfunction in trauma patients.”* Discussion paragraph 1. |
| Limitations | 19 | Discuss limitations of the study, taking into account sources of potential bias or imprecision. Discuss both direction and magnitude of any potential bias  “*However, there are important limitations. The observations in humans are correlative in nature. The degree to which the mechanistic studies in mice translate back to the human condition is uncertain. Because the preponderance of severely injured patients are male, we chose to include only male mice in this initial study. Future studies should include a comparison between males and females. Finally, we did not show whether the histopathologic changes observed in the lungs of mice correlate with changes in pulmonary function*.” Discussion, paragraph 9. |
| Interpretation | 20 | Give a cautious overall interpretation of results considering objectives, limitations, multiplicity of analyses, results from similar studies, and other relevant evidence  “*We found that CXCR2+ neutrophils expressed more IL-5 than CXCR2- neutrophils after HS/T. Others have shown that CXCR2+ is important for neutrophils to migrate to sites of inflammation. Deletion of CXCR2 leads to less inflammation and lower type 2 cytokines (IL-4 and IL-5) in the lungs during allergic airway inflammation. IL-33-induced CXCR2 upregulation is required for neutrophil activation in a mouse model of asthma mediated by IgE. We did not find a role for IL-33 in regulating the increase in neutrophil numbers in the lung after HS/T. Therefore, other pathways likely contribute to neutrophil influx in the lung after HS/T.*” Discussion, paragraph 8.  “*In summary, our studies demonstrate how reverse translation of human observations into mechanistic experimental models can be used to gain insights into trauma-induced immune dysfunction. This strategy allowed us to identify an unsuspected role for IL-33 mediated activation of ILC2 in the initial inflammatory response in the lung after systemic injury. ILC2 regulate neutrophil function through IL-5, which promotes IL-5 expression in neutrophil and drives lung inflammatory changes. As lung dysfunction is a common and serious problem in patients experiencing hemorrhagic shock and tissue trauma, the findings may have identified a novel contributing pathway to this pathologic process that could be targeted through blocking IL-33, IL-5 or their receptors.*” Discussion, paragraph 10. |
| Generalisability | 21 | Discuss the generalisability (external validity) of the study results  Not applicable |
| Other information | | |
| Funding | 22 | Give the source of funding and the role of the funders for the present study and, if applicable, for the original study on which the present article is based  This work was supported by National Institutes of Health grant P50-GM-53789. |
