# Supplementary material for: IL33-mediated ILC2 activation and neutrophil IL5 production in the lung response after severe trauma: A reverse translation study from a human cohort to a mouse trauma model
Source: PLoS Med. 2017 Jul 25;14(7):e1002365. doi: 10.1371/journal.pmed.1002365 (PMC5526517; doi:10.1371/journal.pmed.1002365)
Supplement: S2 Text — The prospective analysis plan was submitted as part of our IRB application on November 2015. (DOCX) [file pmed.1002365.s010.docx]

**PROSPECTIVE ANALYSIS PLAN**

Patients who are severely injured and admitted to the Intensive care unit will be identified based on the following inclusion and exclusion criteria:

**Inclusion Criteria:** Blunt trauma injury- Intact cervical spinal cord-Patient arrives at our hospital ≤2 hours of injury-Admission to ICU and one of the following:-lactate ≥ 2.5 OR -base deficit ≥ 3 OR-hypotension SBP <90 mmHg measured pre-hospital or in trauma bay OR -Receiving blood in trauma bay or pre-hospital OR -requirement for rapid transport to OR with trauma for Ex-lap or thoracic injury repair. For the healthy volunteer group:-Age > 18-Weight > 110 pounds-no recent illness or infection in the last two weeks-no recent hospitalization or trauma in the last month

**Exclusion Criteria:** Age <18; Anticipated survival < 24 hrs; Isolated Traumatic Brain injury (GCS ≤8 after ICU admission) AND brain CT abnormality within 12 hrs of injury; Pregnancy; Penetrating injury; Pre-existing immunosuppression; Transplant recipient; Chronic high doses of steroids (>20 mg prednisone equivalents/day); Known HIV positive status and CD4 count < 200 cells/mm3 Admission to the ICU primary for substance withdrawal. Inability to obtain 1st blood sample within 24 hours of injury. For healthy volunteers: wt < 110 pounds; age < 18; infection or illness in the last two weeks; Hospitalization or trauma in the last month

Blood samples will be collected by venipuncture or from existing intravenous catheter or arterial line placed during routine medical care, whenever possible. Blood draws will be done at the time of study eligibility (within 6 hours of injury) with additional two blood draws within the first 24 hours and then daily thereafter up to day 7 post-injury. The volume of blood to be drawn for the initial draw is approximately 30 cc and approximately 20 cc for the consecutive blood draws. All standard precautions will be undertaken to assure minimal risk, i.e., aseptic technique. Plasma will be separated and frozen at -80 °C for measurement of inflammatory mediators (cytokines and chemokines). The separation and freezing will be done at the research lab. Thus, the maximum blood volume collected over the 7 day period, if all samples are obtained, is approximately 210 cc. For all subjects, demographic data, pre-existing conditions, injury data, biochemical and physiological data, and indices of daily ICU or organ function will be collected from the medical record.

In order to characterize the host systemic inflammatory response to injury, we will measure plasma levels of a broad panel of inflammatory mediators including interleukin (IL)-1β, IL-1 receptor antagonist (IL-1RA), IL-2, soluble IL-2 receptor-α (sIL-2Rα), soluble ST2 (sST2), IL-4, IL-5, IL-6, IL-7, IL-8 (CCL8), IL-9, IL-10, IL-13, IL-15, IL-17A, IL-17E/IL-25, IL-21, IL-22, IL-23, IL-33, interferon (IFN)-γ, IFN-γ inducible protein (IP)-10 (CXCL10), monokine induced by gamma interferon (MIG; CXCL9), macrophage inflammatory protein (MIP)-1α (CCL3), MIP-1β (CCL4), monocyte chemotactic protein (MCP)-1 (CCL2), granulocyte-macrophage colony stimulating factor (GM-CSF), Eotaxin (CCL11), and tumor necrosis factor alpha (TNF-α). We will also measure active and latent TGF-β1 as well nitric oxide reaction products (NO_2_^-^/NO_3_^-^). On Days 0-7, plasma will be separated for measurement of cytokines (as listed above) using the the human inflammatory MILLIPLEX ™ MAP Human Cytokine/Chemokine Panel-Premixed 26 Plex, MILLIPLEX ™ MAP Human Th17 Panel (Millipore Corporation, Billerica, MA) and Luminex™ 100 IS analyzer (Luminex, Austin, TX). Active and latent TGF-β1 will be assessed using a specific ELISA kit (R&D Systems, Minneapolis, MN). NO_2_^-^/NO_3_^-^ will be assayed using a specific kit (Cayman Chemical, San Diego, CA). Clinical markers will include hospital length of stay, ICU length of stay, days on mechanical ventilation, injury severity score, APACHE III scores, mechanism of injury, and mortality.

All data will be analyzed using SigmaPlot™ 11 software (Systat Software, Inc., San Jose, CA) and Graphpad PRISM (GraphPad Software, Inc., La Jolla, CA). Statistical difference between sub-groups will be determined by either Student’s *t*-Test or Fisher’s exact test, as appropriate. Group-time interaction of plasma inflammatory mediators’ levels was determined by One- or Two-Way Analysis of Variance (ANOVA). To quantify the overall production of the statistically significant mediators, we will calculate the area under the curve (AUC) using the mean values for each time point in a given time frame, then calculating the fold change difference between the study groups. *P* < 0.05 will be considered statistically significant for all analyses.
