# Supplementary material for: IL33-mediated ILC2 activation and neutrophil IL5 production in the lung response after severe trauma: A reverse translation study from a human cohort to a mouse trauma model
Source: PLoS Med. 2017 Jul 25;14(7):e1002365. doi: 10.1371/journal.pmed.1002365 (PMC5526517; doi:10.1371/journal.pmed.1002365)

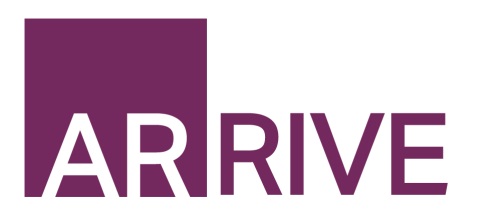


The ARRIVE Guidelines Checklist

Animal Research: Reporting In Vivo Experiments

Carol Kilkenny^1^, William J Browne^2^, Innes C Cuthill^3^, Michael Emerson^4^ and Douglas G Altman^5^

*^1^The National Centre for the Replacement, Refinement and Reduction of Animals in Research, London, UK, ^2^School of Veterinary Science, University of Bristol, Bristol, UK, ^3^School of Biological Sciences, University of Bristol, Bristol, UK, ^4^National Heart and Lung Institute, Imperial College London, UK, ^5^Centre for Statistics in Medicine, University of Oxford, Oxford, UK.*

|  | | ITEM | RECOMMENDATION | Section/ Paragraph |
| --- | --- | --- | --- | --- |
| 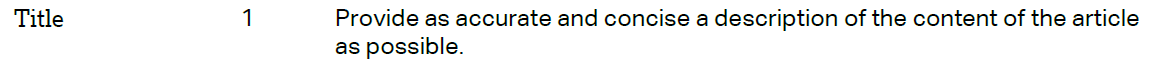 | | | Title |  |
| 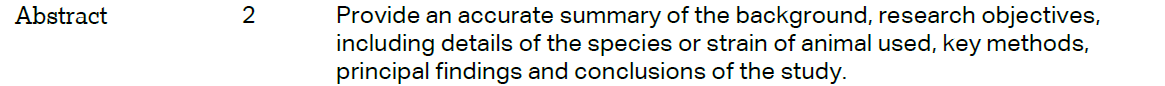 | | | Abstract/1-4 |  |
| INTRODUCTION | | |  |  |
| 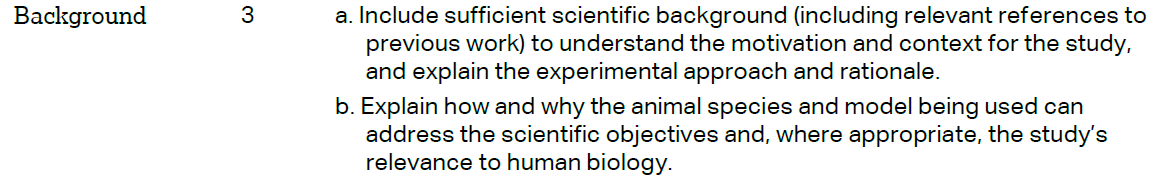 | | | Introduction/4 |  |
| 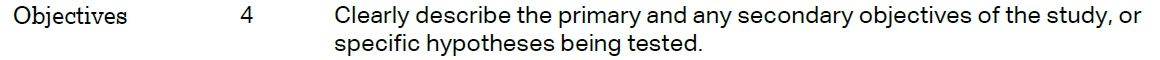 | | | Introduction/4 |  |
| METHODS | | |  |  |
| 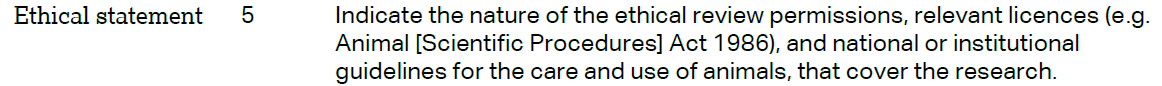 | | | Methods/3 |  |
| 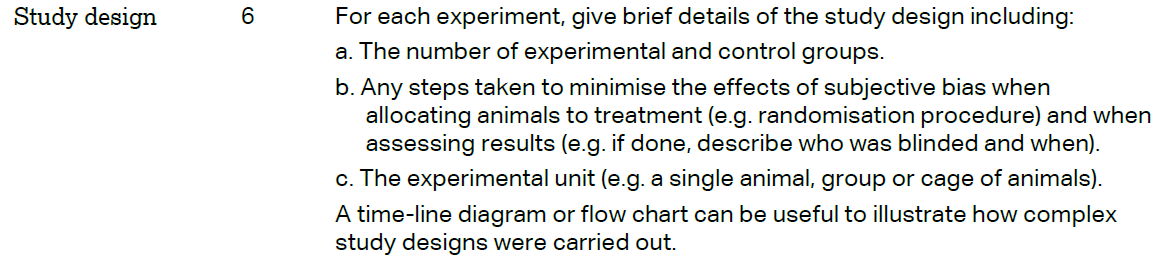 | | | Methods/4,6,9 Legends |  |
| 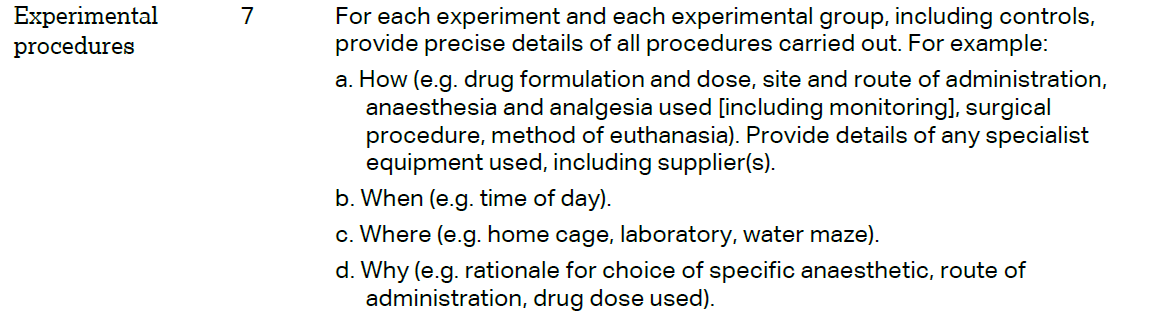 | | | Methods/4 |  |
| 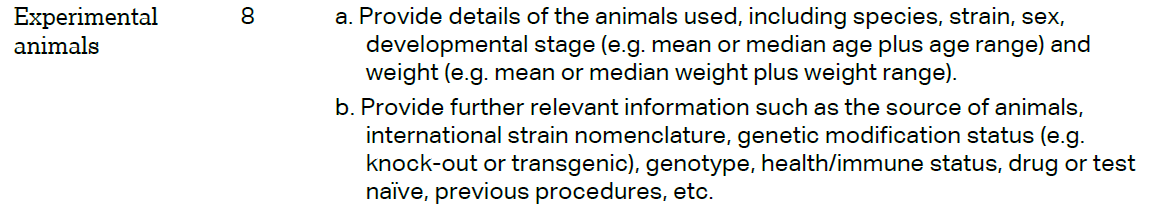 | | | Methods/3,4,6 |  |

The ARRIVE guidelines. Originally published in *PLoS Biology*, June 2010^1^

| 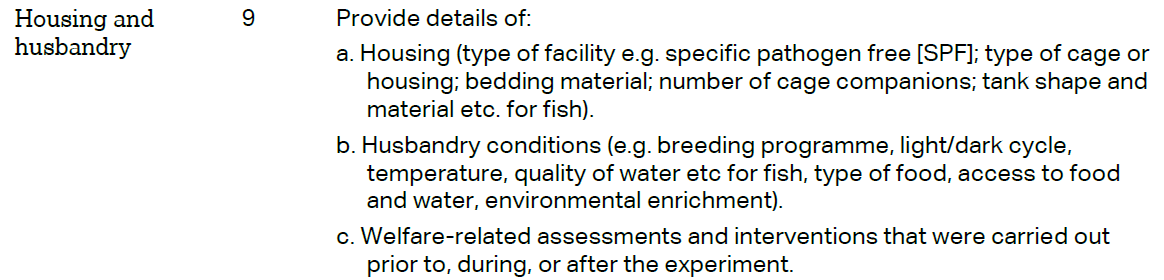 | Methods/3,4 | |
| --- | --- | --- |
| 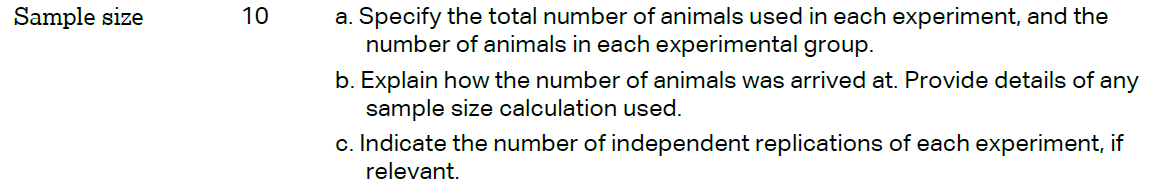 | Legends | |
| 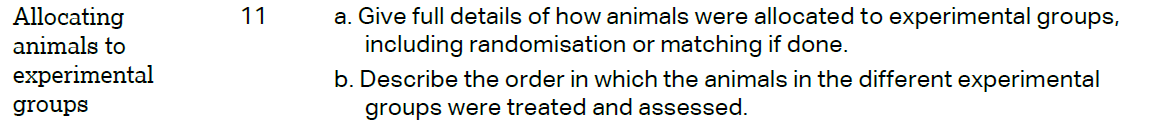 | Methods/4 | |
| 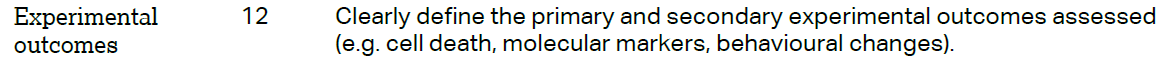 | Methods/5,7,8,9 | |
| 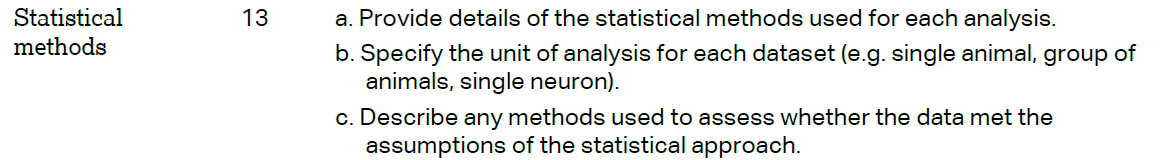 | Methods/10 | |
| RESULTS |  | |
| 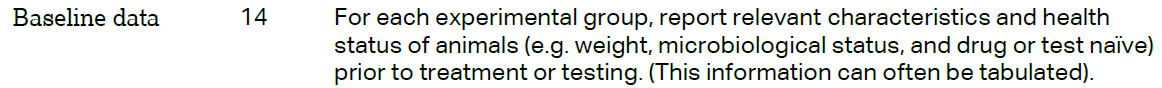 | Methods/4 | |
| 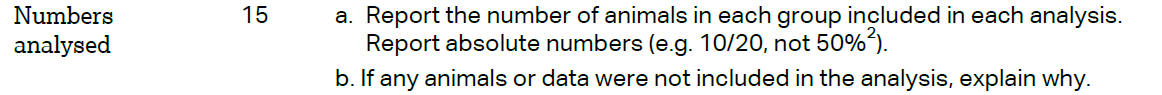 | Legends | |
| 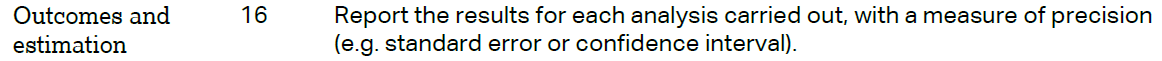 | Result Figures Legends | |
| 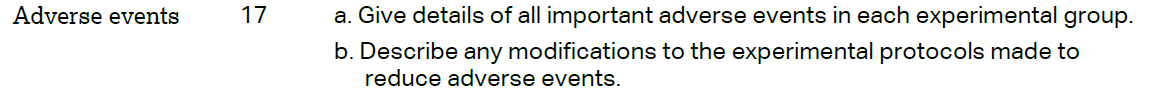 | Methods/4 | |
| DISCUSSION |  | |
| 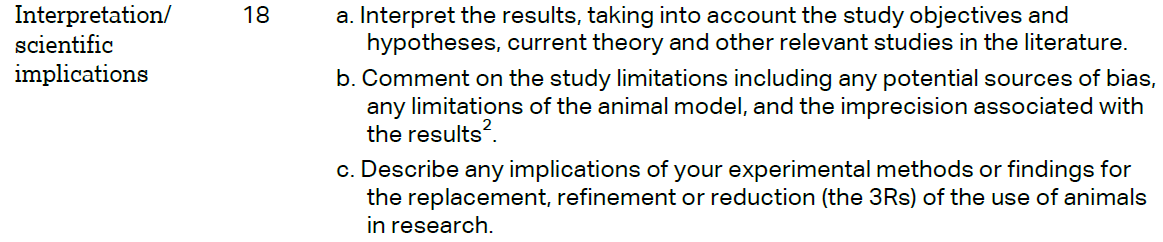 | Discussion/1,4,9 | |
| 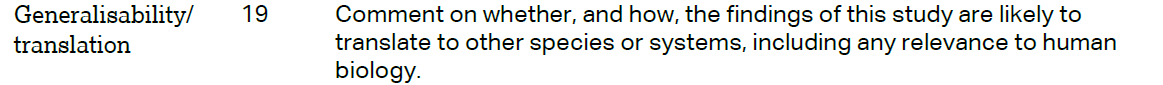 | Discussion/1 | |
| 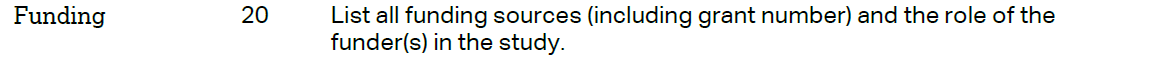 | | End of discussion |


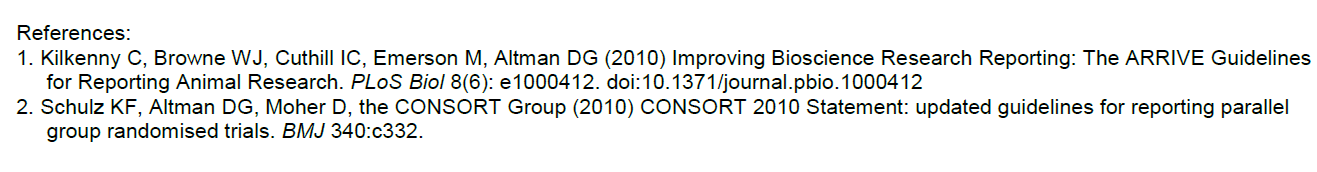

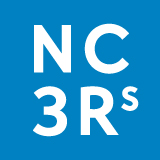

Supplement: S3 Text — (DOCX) [file pmed.1002365.s011.docx]
